# Genome-wide mapping and allelic fingerprinting provide insights into the genetics of resistance to wheat stripe rust in India, Kenya and Mexico

**DOI:** 10.1038/s41598-020-67874-x

**Published:** 2020-07-02

**Authors:** Philomin Juliana, Ravi Prakash Singh, Julio Huerta-Espino, Sridhar Bhavani, Mandeep S. Randhawa, Uttam Kumar, Arun Kumar Joshi, Pradeep Kumar Bhati, Hector Eduardo Villasenor Mir, Chandra Nath Mishra, Gyanendra Pratap Singh

**Affiliations:** 1 International Maize and Wheat Improvement Center (CIMMYT), Texcoco, Mexico; 2 Campo Experimental Valle de Mexico, Instituto Nacional de Investigaciones Forestales, Agricolas Y Pecuarias (INIFAP), Chapingo, Mexico; 3 International Maize and Wheat Improvement Center (CIMMYT), Nairobi, Kenya; 4 CIMMYT, NASC Complex, New Delhi, India; 5 Borlaug Institute for South Asia (BISA), New Delhi, India; 6 ICAR-IIWBR, Agrasain Marg, Karnal, India

**Keywords:** Agricultural genetics, Genetic association study, Genetic markers, Genomics, Plant breeding, Plant genetics

## Abstract

Stripe or yellow rust (YR) caused by *Puccinia striiformis* Westend. f. sp. *tritici* Erikss. is a persistent biotic-stress threatening global wheat production. To broaden our understanding of the shared genetic basis of YR resistance across multi-site and multi-year evaluations, we performed a large genome-wide association study using 43,706 YR observations on 23,346 wheat lines from the International Maize and Wheat Improvement Center evaluated between 2013 and 2019 at sites in India, Kenya and Mexico, against predominant races prevalent in the countries. We identified 114 repeatable markers tagging 20 quantitative trait loci (QTL) associated with YR on ten chromosomes including 1D, 2A, 2B, 2D, 3A, 4A, 4D, 5A, 5B and 6B, among which four QTL, *QYr.cim-2DL.2*, *QYr.cim-2AS.1*, *QYr.cim-2BS.2* and *QYr.cim-2BS.3* were significant in more than ten datasets. Furthermore, we report YR-associated allelic fingerprints for the largest panel of wheat breeding lines (52,067 lines) till date, creating substantial opportunities for YR favorable allele enrichment using molecular markers. Overall, the markers and fingerprints reported in this study provide excellent insights into the genetic architecture of YR resistance in different geographical regions, time-periods and wheat germplasm and are a huge resource to the global wheat breeding community for accelerating YR resistance breeding efforts.

## Introduction

The basidiomycete fungus *Puccinia striiformis* Westend. f. sp. *tritici* Erikss. (*Pst*) that causes stripe rust or yellow rust (YR) in common wheat (*Triticum aestivum* L.) has been a significant threat to global food security^1,2^. The pathogen is prevalent in more than 60 countries^3,4^, and an estimated 88% of the world’s wheat production is considered vulnerable^5^. While yield reductions due to YR range between 5 and 50%, losses up to 100% on highly susceptible cultivars are possible^3,6^. In the past, several severe YR epidemics have been reported^5,7–10^, that were generally driven by changing weather patterns, favorable weather conditions, the emergence of novel aggressive *Pst* strains that overcome resistance genes, the adaptation of strains that were initially confined to regions with cool and wet climates to warmer climates, highly divergent genetic lineages of races, etc.^6,9,11–13^. Management of YR is possible through the appropriate use of fungicides and cultural practices, but the deployment of resistant cultivars^2^ is considered to be the most cost-effective, environmentally safe and sustainable strategy.

Genetic resistance to YR based on the race-specificity and growth stage can be classified into: race-specific seedling or all-stage resistance and race non-specific adult-plant resistance (APR)^14–16^. Seedling resistance detected in the seedling stage, is often expressed at all stages, exhibits race-specificity, and can be quickly overcome by new *Pst* races^17–21^. Meanwhile, APR is expressed at the later growth stages, is usually durable and not race-specific^16,22^. In addition, resistance to YR can also be classified based on the testing conditions into: greenhouse and field consistent resistance that is usually detected in the greenhouse in seedlings and is also effective in the field (henceforth referred to as seedling resistance, SR) and field resistance (FR) that can be detected at the adult-plant stage in the field. Among the 83 catalogued YR resistance genes^23,24^, most of them are all-stage resistance genes with the exception of 22 genes namely *Yr11-14, Yr16, Yr18/Lr34/Sr57/Pm38/Ltn1, Yr29/Lr46/Sr58/Pm39/Ltn2, Yr30/Lr27/Sr2, Yr36, Yr39, Yr46/Lr67/Sr55/Pm46/Ltn3, Yr52, Yr59, Yr62, Yr68, Yr71, Yr75, Yr77-80* and *Yr82*^9,15,25–27^. In addition, several temporary YR resistance genes and quantitative trait loci (QTL) have been identified and reviewed^9,28^.

A key component in developing YR resistant wheat varieties involves identifying genes/QTL and closely linked diagnostic markers that can facilitate accurate selection for resistance. While linkage mapping has been very useful for *Yr* gene identification, genome-wide association studies (GWAS) that rely on the linkage disequilibrium (LD) between markers and the underlying causal polymorphisms are powerful for identifying marker-trait associationsl^29–31^. In contrast to linkage mapping studies, GWAS involve no population development time as they can be performed on existing diversity panels and also provide better resolution by harnessing ancestral recombination events that have occurred at the population level in a diversity panel^29,32^. While several GWAS for YR have been reported^33–39^, our understanding of the shared genetic basis of YR resistance across multi-site and multi-year evaluations is inadequate and several marker-trait associations identified in GWAS are not repeatable, limiting their use in breeding programs. Breeding for YR was initiated at the International Maize and Wheat Improvement Center (CIMMYT) in the early 1970’s, and involves crossing parents with slow rusting APR genes, selecting segregating early-generations under high YR pressure^40^ in Toluca (Mexico), and screening advanced generations in Karnal (India), Ludhiana (India), Njoro (Kenya), Celaya (Mexico), El Batan (Mexico) and Toluca (Mexico). Between 2013 and 2019, 23,346 wheat breeding lines have been evaluated for YR by CIMMYT and 43,706 YR observations have been recorded at sites in India, Kenya and Mexico. Hence, the key objective of this study was to leverage these datasets and perform GWAS for dissecting the shared genetic basis of YR FR against the races predominant in these three countries.

Another critical component in developing YR resistant wheat varieties involves identifying the best parental combinations with a high number of favorable alleles (FAs) for YR associated genes/QTL. So, we have generated the allelic fingerprints of a huge panel of 52,067 CIMMYT wheat breeding lines comprising all the advanced lines developed during 2013–2019 and key parents in the crossing blocks for repeatable markers significantly associated with YR resistance. Furthermore, we used the allelic fingerprint profiles to gain insights into the following questions: (i) What is the proportion of YR FAs in the CIMMYT germplasm? (ii) Which parents are the most likely sources of resistance and what are the lines with high frequencies of FAs that can be selected as potential parents? (iii) What is the effect of having different numbers of QTL with FAs on the disease severity?

## Results

### Phenotyping data and population structure analysis

**Figure 1:**
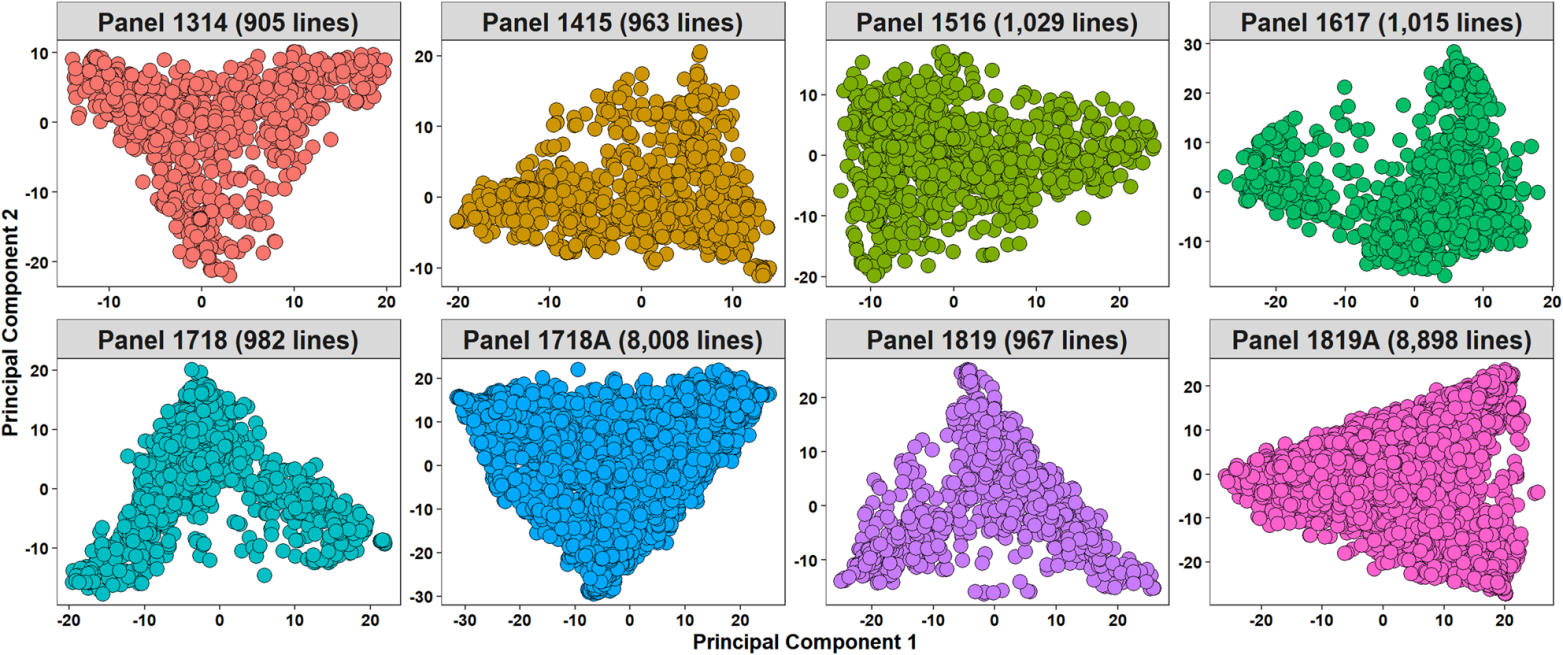
Population structure analysis of the different panels showing the plot of Principal Component 1 (PC1) vs. Principal Component 2 (PC2). The percentage variance explained by PC1 and PC2 were 8% and 6.9% in panel 1314 with 905 lines, 9.6% and 4.5% in panel 1415 with 963 lines, 7.2% and 4.3% in panel 1516 with 1,029 lines, 8.9% and 7.8% in panel 1617 with 1,015 lines, 11.7% and 8.4% in panel 1718 with 982 lines, 8.2% and 6.3% in panel 1718A with 8,008 lines, 12.8% and 7.9% in panel 1819 with 967 lines and 18% and 5.6% in panel 1819A with 8,898 lines. The cumulative percentage variation explained by the first two principal components ranged between 11.5 and 23.6%.

Phenotypic distributions of YR FR and SR are shown in Fig. S1a–S1e and phenotyping data is available in Table S2. Statistical analysis of YR FR data (Table S3) indicated that the highest YR mean percentage severity was in Karnal panel 1819 (22.5 ± 23.5%), followed by Njoro main season (MS) 2 panel 1718 and Ludhiana panels 1415, 1819 and 1718 all of which had mean severities greater than 15%. Considering SR, the highest mean of the infection scores was observed in panel 1516 (6.5 ± 1.4) and the least in panel 1819 (3.8 ± 1.1). In Njoro, panels 1314, 1415 and 1516 had mean field severities less than 3.4% and an increasing severity was observed in the recent panels 1718 and 1819. Population structure analysis in the eight panels using the first two principal components indicated weak to moderate population structure, with the cumulative percentage variation explained by the first two principal components ranging between 11.5 and 23.6%. (Fig. 1).

### Genome-wide association mapping for stripe rust resistance

**Figure 2:**
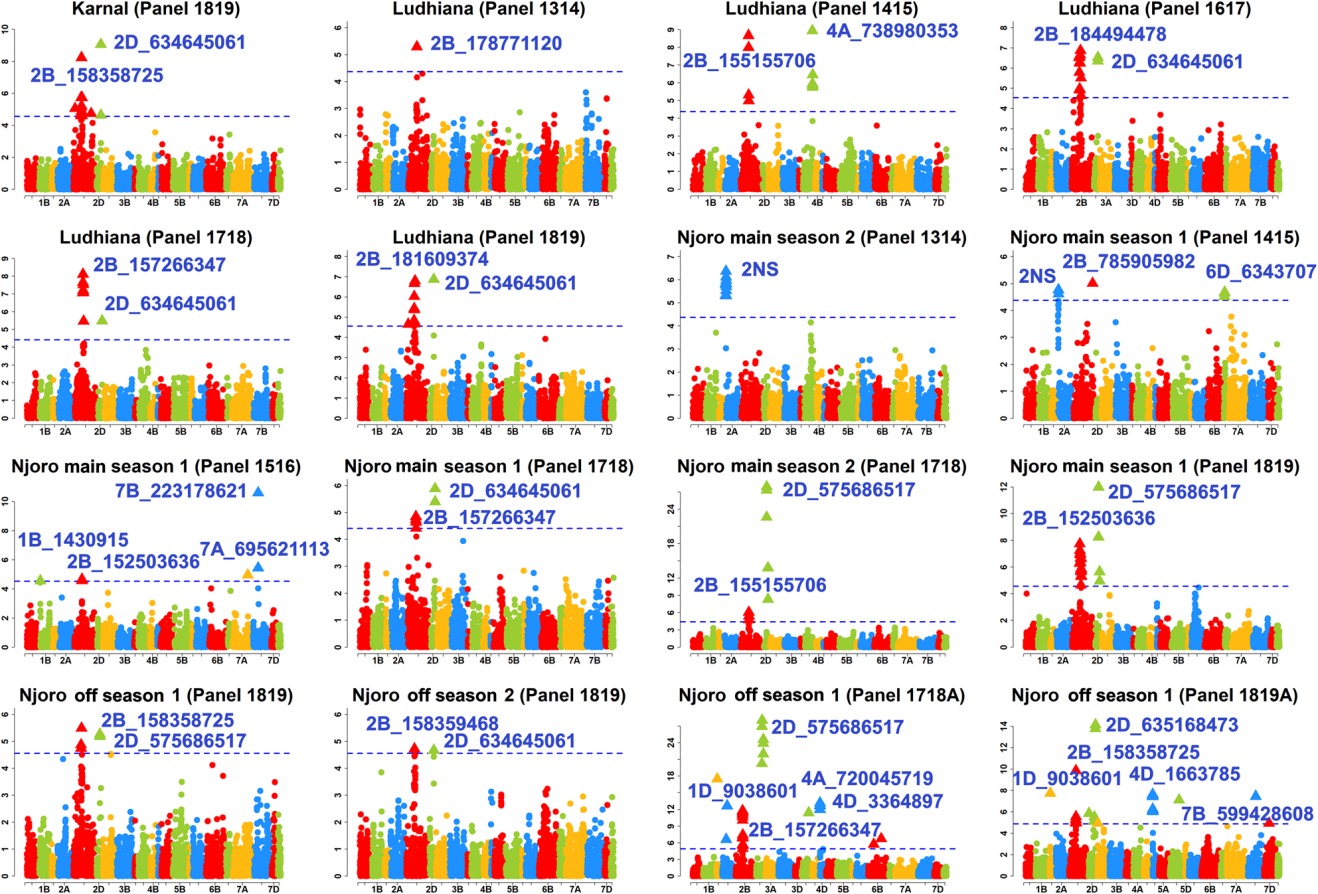
Markers significantly associated with field resistance to stripe rust in Karnal and Ludhiana, India and Njoro Kenya. Panels 1314, 1415, 1516, 1617 and 1718 had 1,092 lines each. Panel 1819 had 1,228 lines, panel 1718A had 8,593 lines and panel 1819A had 9,217 lines. In Njoro, Kenya, the main season refers to the June to October crop cycle and the off season refers to the January to May crop cycle. The threshold lines correspond to the threshold using the Bonferroni correction for multiple testing at an α level of 0.20.

**Figure 3:**
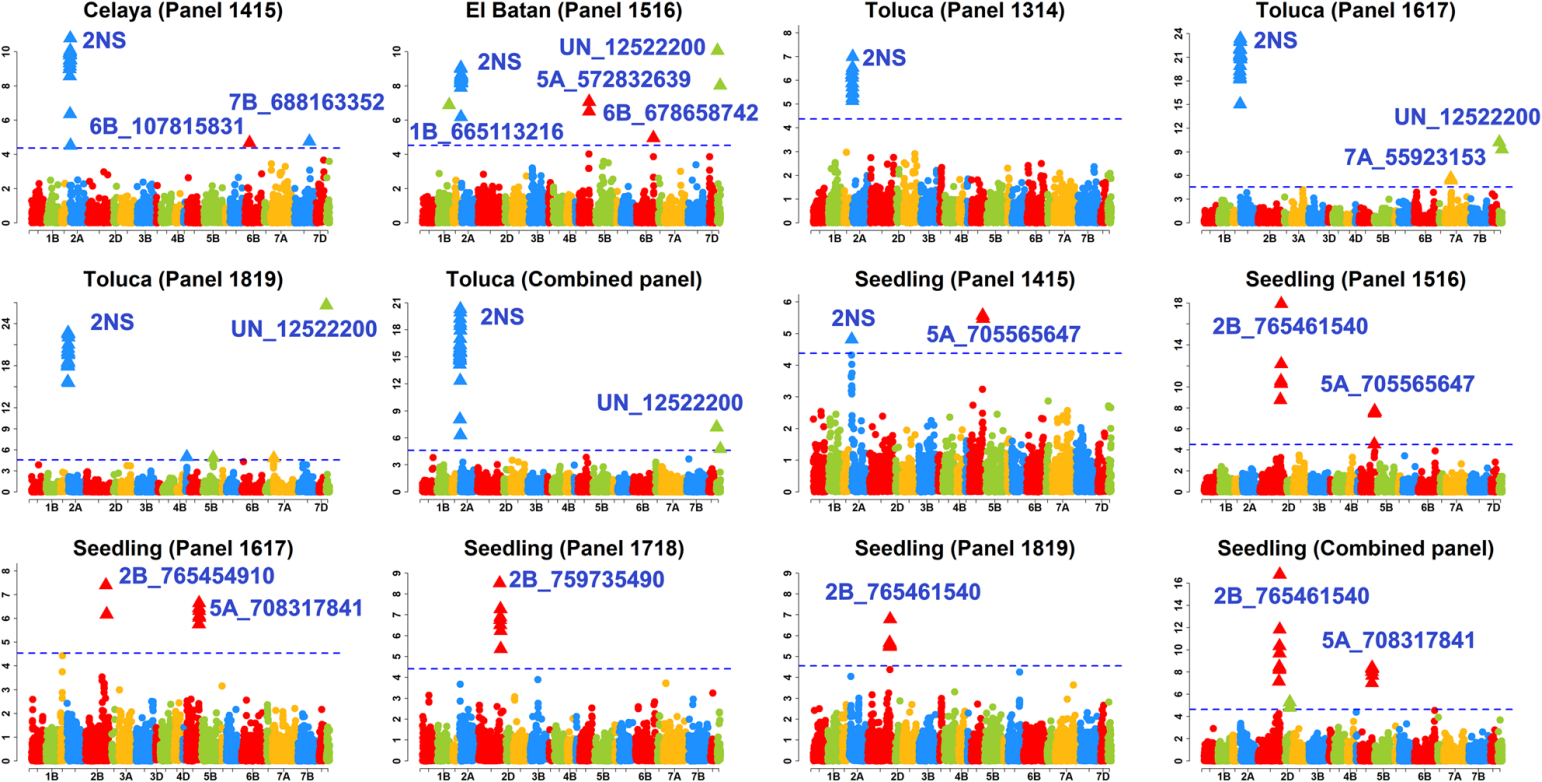
Markers significantly associated with field resistance to stripe rust in Celaya, El Batan and Toluca, Mexico and seedling resistance to stripe rust race Mex14.191. Panels 1314, 1415, 1516, 1617 and 1718 had 1,092 lines each. Panel 1819 had 1,228 lines, the combined panel for Toluca had 3,412 lines and the combined panel for greenhouse resistance had 5,075 lines. The threshold lines correspond to the threshold using the Bonferroni correction for multiple testing at an α level of 0.20.

We identified 466 markers significantly associated with YR resistance at a *p *value threshold of 0.001 across all the datasets. The *p *values, additive effects and the R^2^ of the markers are given in Table S4. After Bonferroni correction for multiple testing, we identified 146 markers that were significantly associated with YR resistance (Figs. 2 and 3, Table S5).

### Markers associated with field resistance in India (Karnal and Ludhiana)

In the Karnal panel 1819, the most significant markers were 2D_634645061, 2D_635168473 and 2B_158358725 that had high additive effects (AEs) of 10.3%, 10.3% and 11.8% on the severity, respectively. In the Ludhiana panel 1314, the most significant marker was 2B_178771120 with an AE of 5.4%. In the Ludhiana panel 1415, 4A_738980353 and 2B_155155706 were the most significant markers with AEs of 7.7%, and 9.2%, respectively. In the Ludhiana panel 1617, the most significant markers on chromosomes 2B and 2D were 2B_184494478 and 2D_634645061 that had AEs of 2.5% and 3.8%, respectively. In the Ludhiana panel 1718, the most significant markers on chromosomes 2B and 2D were 2B_157266347, 2D_634645061 and 2D_635168473 that had AEs of 9.1%, 7.4% and 7.4%, respectively. In the Ludhiana panel 1819, the most significant markers were 2D_634645061, 2D_635168473 and 2B_181609374 that had AEs of 7%, 7% and 5%, respectively. Considering all the datasets in India, the markers that were significant in the highest number of datasets (four out of six) included 2B_155155706, 2D_634645061 and 2D_635168473.

### Markers associated with field resistance in Njoro, Kenya

In the Njoro MS1 panel 1415, the most significant markers were 2B_785905982 and 2A_15449240 that had AEs of 2.4% and 1.5%, respectively. In the Njoro MS1 panel 1516, the most significant marker was 7B_223178621 with a low AE of 0.5%. In the Njoro MS1 panel 1718, the most significant markers on chromosomes 2D and 2B were 2D_634645061, 2D_635168473 and 2B_157266347 that had AEs of 4.3%, 4.3% and 3.8%, respectively. In the Njoro MS1 panel 1819, the most significant markers on chromosomes 2D and 2B were 2D_575686517 and 2B_152503636 that had AEs of 5.2% and 4.9%, respectively. In the Njoro MS2 panel 1314, the most significant marker was 2A_14418760 with an AE of 1.4%. In the Njoro MS2 panel 1718, the most significant marker was 2D_575686517 with a high AE of 13.1%. In the Njoro off season (OS) 1 panel 1819, the most significant markers were 2B_158358725 and 2D_575686517 that had AEs of 3.9% and 1.9%, respectively. In the Njoro OS2 panel 1819, the most significant markers were 2B_158359468 and 2D_634645061 that had AEs of 2.1% and 3.1%, respectively. In the Njoro OS1 panel 1718A, the most significant marker was 2D_575686517 with an AE of 4.1%. In the Njoro OS1 panel 1819A, the most significant marker was 2D_635168473 with an AE of 2.6%. Overall, across all the FR evaluations in Njoro, markers 2D_634645061 and 2D_635168473 were significant in seven datasets.

### Markers associated with field resistance in Mexico (Celaya, El Batan and Toluca)

In the Celaya panel 1415, the most significant marker was 2A_18495181 with an AE of 6.3%. In the El Batan panel 1516, the most significant marker with a known chromosomal position was 2A_2367215 with an AE of 1.5%. In the Toluca panels 1314 and 1617, the most significant markers were 2A_24002740 and 2A_17830617 with AEs of 1.7% and 1.1%, respectively. In the Toluca panel 1819, the most significant marker with a known chromosomal position was 2A_14978553 with an AE of 2.2%. In the Toluca combined panel, the most significant marker was 2A_18495181 with an AE of 2%. Across all the FR evaluations in Mexico, the markers significant in all the six datasets were on chromosome 2A.

### Markers associated with seedling resistance

In the seedling panel 1415, the most significant marker was 5A_705565647 with an AE of 0.6 on the infection score. In the seedling panel 1516, the most significant markers on chromosomes 2B and 5A were 2B_765461540 and 5A_705565647 that had AEs of 0.9 and 0.6, respectively. In the seedling panel 1617, the most significant markers on chromosomes 2B and 5A were 2B_765454910 and 5A_708317841 that had AEs of 0.6 and 0.5, respectively. In the seedling panel 1718, the most significant marker was 2B_759735490 with an AE of 0.6. In the seedling panel 1819, the most significant marker was 2B_765461540 with an AE of 0.5. In the seedling combined panel, the most significant markers on chromosomes 2B and 5A were 2B_765461540 and 5A_708317841 with AEs of 1.7 and 0.5, respectively. The markers significant in the highest number of seedling panels were on chromosomes 2B (five out of six datasets) and 5A (four out of six datasets).

### A reference map with stripe rust resistance associated markers

**Figure 4:**
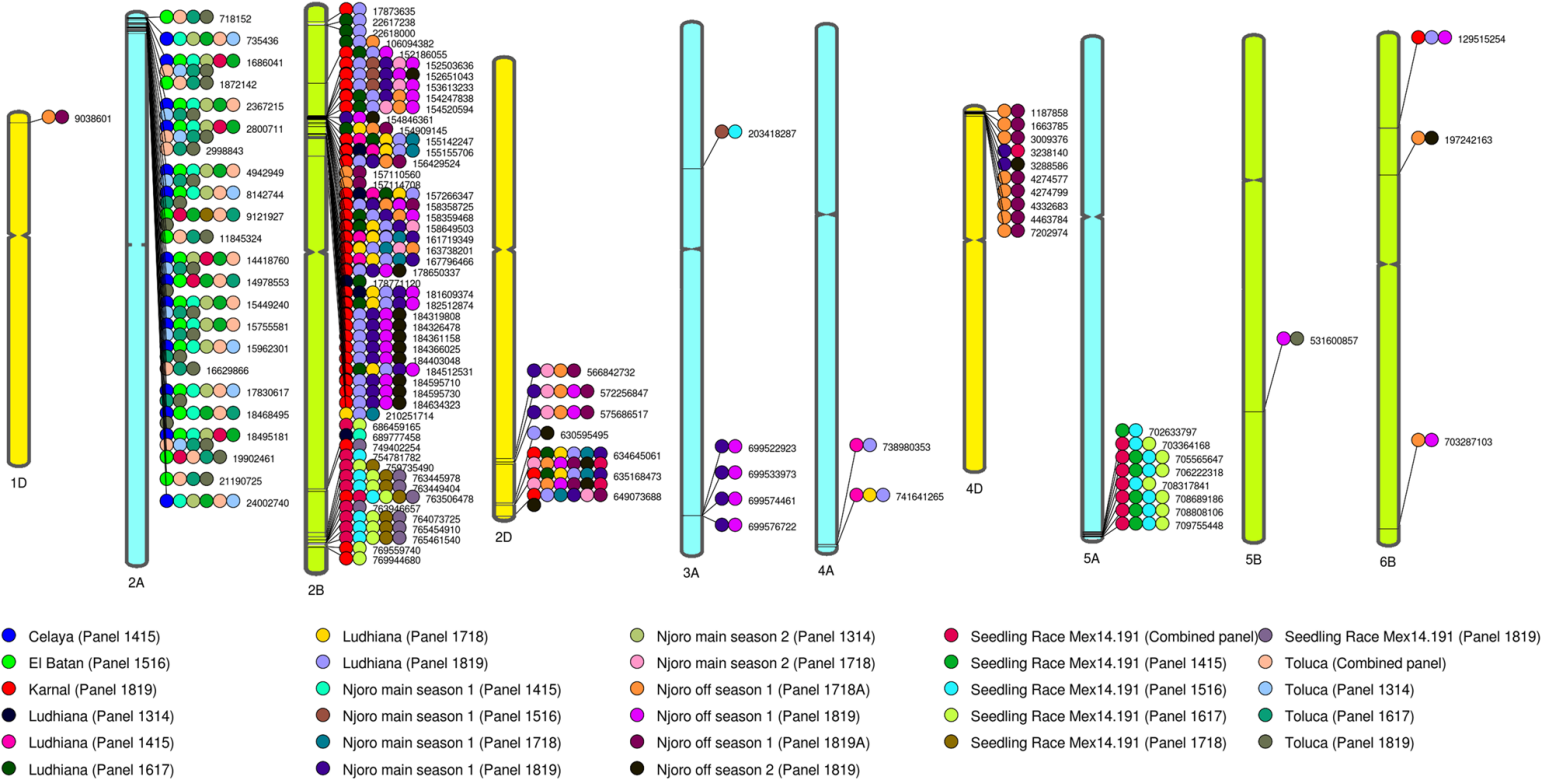
A reference map with stripe rust resistance associated markers significant in two or more datasets.

To identify repeatable markers associated across multiple datasets, we filtered the markers that were significant in two or more datasets at a *p *value threshold of 0.001, resulting in 114 markers. The repeatable markers with known chromosomal positions were anchored to the RefSeq v1.0 and a reference map for YR resistance was generated (Fig. 4). The markers significant in the highest number (12) of datasets were 2D_634645061 and 2D_635168473, followed by markers 2B_157266347, 2B_155142247, 2B_155155706 and several markers on chromosome 2A that were significant in ten to eleven datasets.

### Designation of linkage disequilibrium based quantitative trait loci associated with stripe rust

The LD between the 114 repeatable markers in each chromosome was obtained and used to delineate LD-based QTL (Table S6, Fig. S2a–S2h). A subset of these markers that included the top two significant markers in each dataset and chromosome that were repeatable are shown in Table 1, along with their genetic positions in the POPSEQ map^41^ and the designated QTL. On chromosome 1DS, *QYr.cim-1DS.1* was associated with Njoro OS panels 1718A and 1819A. On chromosome 2AS, *QYr.cim-2AS.1* spanning 8.9 cM had 23 markers that were associated with FR in Njoro MS panels 1314 and 1415, Celaya, El Batan, Toluca all panels, and SR in panels 1415, 1718 and the combined panel. We also observed that the two unaligned markers were in high LD with the markers in *QYr.cim-2AS.1* and indicate the same QTL. On chromosome 2BS, *QYr.cim-2BS.1* was associated with YR FR in the Ludhiana panels 1617 and 1819. In addition, a large region on chromosome 2BS between 152186055 and 184634323 bps spanning 32.4 Mb and 5 cM had markers significantly associated with several datasets. Despite being a narrow genetic interval, several markers in this region had very low correlations with each other, suggesting the possibility of more than one QTL. Hence, we used a stringent marker correlation coefficient (R^2^ > 0.8) and D′ value (D′ > 0.9) to designate QTL in this region, and also classified only the markers that were highly significant in several datasets into QTL. Among these, *QYr.cim-2BS.2* was associated with FR in Karnal, Ludhiana panel 1819, Njoro MS panels 1516, 1718 and 1819 and Njoro OS panel 1819. *QYr.cim-2BS.3* was associated with FR in Karnal, Ludhiana all panels, Njoro MS panels 1718 and 1819 and Njoro OS panels 1718A and 1819A. *QYr.cim-2BS.4* was associated with Karnal, Ludhiana panels 1617, 1718 and 1819, Njoro MS panels 1718 and 1819 and Njoro OS panels 1819, 1718A and 1819A. *QYr.cim-2BS.5* was associated with Karnal, Ludhiana panels 1314, 1617, 1718 and 1819, Njoro MS and OS panel 1819.

**Table 1:** Markers significantly associated with stripe rust field resistance in India, Kenya, Mexico and greenhouse resistance in two or more datasets, their positions, *p* values for the test of significance, additive effects, percentage variation explained, their designated linkage-disequilibrium based quantitative trait loci (QTL) and the percentage of lines with favorable alleles (FAs) at the QTL.

Marker	Chromo-some	Physical position (bps)	Genetic position (cM)	*p *value	Additive effect	Percentage variation explained (%)	Dataset	Designated QTL	Percentage of lines with FAs at the QTL (Number of FAs/Number of non-missing alleles)
1D_9038601	1DS	9038601	11	3.27E−18	6.65	1.06	Njoro OS1 panel 1718A	*QYr.cim-1DS.1*	2.8% (1,275/45,088)
				1.79E−08	2.41	0.40	Njoro OS1 panel 1819A		
2A_9121927	2AS	9121927	8.9	2.10E−04	0.53	1.76	Seedling panel 1718	*QYr.cim-2AS.1*	79.3% (41,293/52,067)
				4.75E−05	0.49	3.66	Seedling panel 1415		
2A_14418760	2AS	14418760	8.9	4.28E−07	1.39	3.29	Njoro MS2 panel 1314		
				4.15E−04	0.42	0.34	Seedling combined panel		
				1.52E−05	0.64	4.08	Seedling panel 1415		
2A_14978553	2AS	14978553	8.9	8.54E−24	1.14	11.20	Toluca panel 1617		
				1.92E−23	2.20	11.46	Toluca panel 1819		
2A_15449240	2AS	15449240	8.9	1.70E−05	1.52	2.31	Njoro MS1 panel 1415		
				7.52E−07	1.53	3.16	Njoro MS2 panel 1314		
2A_17830617	2AS	17830617	8.9	2.41E−05	1.59	2.24	Njoro MS1 panel 1415		
				4.06E−24	1.10	11.36	Toluca panel 1617		
2A_18495181	2AS	18495181	8.9	1.64E−11	6.34	5.32	Celaya panel 1415		
				4.89E−21	1.99	3.21	Toluca combined panel		
				2.91E−07	1.67	3.41	Toluca panel 1314		
				2.79E−23	2.17	11.38	Toluca panel 1819		
2B_152503636	2BS	152503636	77.7	2.19E−05	1.01	2.15	Njoro MS1 panel 1516	*QYr.cim-2BS.2*	68.9% (31,980/46,417)
				1.81E−08	4.88	4.02	Njoro MS1 panel 1819		
				1.34E−05	2.32	2.51	Njoro OS1 panel 1819		
2B_153613233	2BS	153613233	77.7	2.56E−05	1.02	2.12	Njoro MS1 panel 1516		
				5.80E−08	4.82	3.75	Njoro MS1 panel 1819		
2B_155142247	2BS	155142247	77.7	2.17E−09	9.23	4.24	Ludhiana panel 1415	*QYr.cim-2BS.3*	10.6% (5,479/51,732)
				2.09E−12	5.03	0.71	Njoro OS1 panel 1718A		
2B_155155706	2BS	155155706	77.7	2.17E−09	9.23	4.24	Ludhiana panel 1415		
				2.21E−08	9.11	3.66	Ludhiana panel 1718		
				1.44E−05	3.93	2.30	Njoro MS1 panel 1718		
				8.48E−07	11.81	2.95	Njoro MS2 panel 1718		
2B_157266347	2BS	157266347	77.7	7.72E−09	9.06	3.88	Ludhiana panel 1718		
				1.41E−05	3.80	2.31	Njoro MS1 panel 1718		
				1.64E−12	5.08	0.71	Njoro OS1 panel 1718A		
				1.57E−07	4.85	3.52	Ludhiana panel 1819		
2B_765454910	2BL	765454910	134	1.44E−12	0.44	1.21	Seedling combined panel	*QYr.cim-2BL.2*	14.1% (7,337/52,005)
				6.59E−13	0.66	5.63	Seedling panel 1516		
				8.85E−12	0.61	5.27	Seedling panel 1617		
2B_765461540	2BL	765461540	134	1.66E−17	0.65	1.72	Seedling combined panel		
				1.21E−18	0.92	8.39	Seedling panel 1516		
				2.07E−11	0.68	5.09	Seedling panel 1617		
				1.61E−07	0.49	3.49	Seedling panel 1819		
2D_572256847	2DL	572256847	82.4	1.05E−12	5.19	6.28	Njoro MS1 panel 1819	*QYr.cim-2DL.1*	30% (14,395/48,053)
				4.79E−28	13.10	13.96	Njoro MS2 panel 1718		
				1.10E−27	4.05	1.64	Njoro OS1 panel 1718A		
				6.59E−06	1.87	2.67	Njoro OS1 panel 1819		
2D_575686517	2DL	575686517	82.4	1.03E−12	5.18	6.29	Njoro MS1 panel 1819		
				1.32E−28	13.12	14.27	Njoro MS2 panel 1718		
				8.67E−29	4.12	1.71	Njoro OS1 panel 1718A		
				5.40E−06	1.91	2.71	Njoro OS1 panel 1819		
2D_634645061	2DL	634645061	101.1	8.64E−10	10.30	4.71	Karnal panel 1819	*QYr.cim-2DL.2*	5.3% (2,648/50,079)
				2.94E−07	3.75	3.01	Ludhiana panel 1617		
				3.27E−06	7.38	2.61	Ludhiana panel 1718		
				1.32E−07	7.01	3.56	Ludhiana panel 1819		
				1.30E−06	4.31	2.80	Njoro MS1 panel 1718		
				1.64E−14	2.59	0.72	Njoro OS1 panel 1819A		
				2.14E−05	3.05	2.26	Njoro OS2 panel 1819		
				5.11E−06	0.36	0.54	Seedling combined panel		
2D_635168473	2DL	635168473	101.1	8.64E−10	10.31	4.71	Karnal panel 1819		
				4.37E−07	3.65	2.93	Ludhiana panel 1617		
				3.27E−06	7.38	2.61	Ludhiana panel 1718		
				1.32E−07	7.01	3.56	Ludhiana panel 1819		
				1.30E−06	4.31	2.80	Njoro MS1 panel 1718		
				7.36E−15	2.61	0.74	Njoro OS1 panel 1819A		
				2.14E−05	3.05	2.26	Njoro OS2 panel 1819		
				1.57E−05	0.34	0.49	Seedling combined panel		
3A_699522923	3AL	699522923	131.9	1.28E−04	0.69	2.00	Njoro MS1 panel 1819	*QYr.cim-3AL.2*	13.4% (5,858/43,635)
				2.90E−05	0.25	2.33	Njoro OS1 panel 1819		
4A_738980353	4AL	738980353	134	1.15E−09	7.70	4.37	Ludhiana panel 1415	*QYr.cim-4AL.1*	15.9% (7,590/47,772)
				2.81E−04	4.64	1.82	Ludhiana panel 1819		
4A_741641265	4AL	741641265	140.5	1.13E−06	6.09	2.89	Ludhiana panel 1415		
				3.97E−04	4.89	1.61	Ludhiana panel 1718		
				2.23E−04	4.74	1.88	Ludhiana panel 1819		
4D_1663785	4DS	1663785	–	2.34E−13	5.62	0.77	Njoro OS1 panel 1718A	*QYr.cim-4DS.1*	2.7% (1,287/47,458)
				2.31E−08	2.28	0.40	Njoro OS1 panel 1819A		
4D_3238140	4DS	3238140	–	9.13E−04	1.56	1.56	Njoro MS1 panel 1819	*QYr.cim-4DS.2*	32.7% (15,572/47,661)
				4.09E−05	0.19	0.45	Seedling combined panel		
4D_3288586	4DS	3288586	–	5.18E−04	1.53	1.69	Njoro MS1 panel 1819		
				7.34E−04	1.14	1.51	Njoro OS2 panel 1819		
5A_705565647	5AL	705565647	128	2.68E−06	0.64	4.74	Seedling panel 1415	*QYr.cim-5AL.1*	9.8% (5,101/51,918)
				1.93E−08	0.59	3.53	Seedling panel 1516		
5A_706222318	5AL	706222318	128	2.68E−06	0.64	4.74	Seedling panel 1415		
				2.16E−08	0.59	3.51	Seedling panel 1516		
				3.69E−07	0.49	3.04	Seedling panel 1617		
5A_708317841	5AL	708317841	128	3.89E−09	0.49	0.86	Seedling combined panel		
				2.22E−07	0.51	3.14	Seedling panel 1617		
5B_531600857	5BL	531600857	86	9.40E−04	2.29	1.55	Njoro OS1 panel 1819	*QYr.cim-5BL.1*	82.5% (40,635/49,245)
				1.28E−04	0.26	1.88	Toluca panel 1819		
6B_129515254	6BS	129515254	52	6.46E−04	2.54	1.63	Karnal panel 1819	*QYr.cim-6BS.1*	94.2% (40,743/43,246)
				1.16E−04	1.41	2.02	Ludhiana panel 1819		
				7.45E−05	0.63	2.12	Njoro OS1 panel 1819		
6B_197242163	6BS	197242163	65	4.08E−04	3.41	0.20	Njoro OS1 panel 1718A		
				5.68E−04	3.05	1.57	Njoro OS2 panel 1819		
6B_703287103	6BL	703287103	135	6.14E−04	1.70	0.19	Njoro OS1 panel 1718A	*QYr.cim-6BL.2*	80.4% (36,141/44,928)
				1.90E−04	2.39	1.91	Njoro OS1 panel 1819		

On chromosome 2BL, *QYr.cim-2BL.1* was associated with FR in the Ludhiana panel 1314 and Njoro MS panel 1415, and SR in panel 1617 and the combined panel. Another QTL on chromosome 2BL, *QYr.cim-2BL.2* was associated with FR in Karnal, SR in panels 1516, 1617, 1718, 1819 and in the combined panel. On chromosome 2DL, *QYr.cim-2DL.1* was associated with YR FR in Njoro MS panels 1718 and 1819 and in the OS panels 1819, 1718A and 1819A. In addition, *QYr.cim-2DL.2* was associated with FR in Karnal, Ludhiana panels 1617, 1718 and 1819, Njoro MS panels 1718 and 1819, Njoro OS panels 1718A, 1819A, 1819 and SR in the combined panel. On chromosome 3AS, *QYr.cim-3AS.1* was associated in with YR FR in Njoro MS and SR in panel 1516. On chromosome 3AL, *QYr.cim-3AL.1* at 131.9 cM was associated with YR FR in the Njoro MS and OS in panel 1819. On chromosome 4AL, *QYr.cim-4AL.1* was associated with YR FR in Ludhiana panels 1415, 1718 and 1819. On chromosome 4DS, *QYr.cim-4DS.1* was associated with YR FR in the Njoro OS panels 1718A and 1819A. *QYr.cim-4DS.2* was associated with FR in the Njoro MS and OS panel 1819, in addition to SR in the combined panel. On chromosome 5AL, *QYr.cim-5AL.1* was associated with SR to YR in panels 1415, 1516, 1617 and the combined panel. On chromosome 5BL, *QYr.cim-5BL.1* was associated with Njoro OS and Toluca panel 1819. On chromosome 6BS, *QYr.cim-6BS.1* was associated with Karnal, Ludhiana panel 1819, Njoro OS panels 1819 and 1718A. Finally, on chromosome 6BL, *QYr.cim-6BL.1* was associated with resistance in the Njoro OS panels 1718A and 1819.

### Allelic fingerprinting of lines for stripe rust resistance associated markers

**Figure 5:**
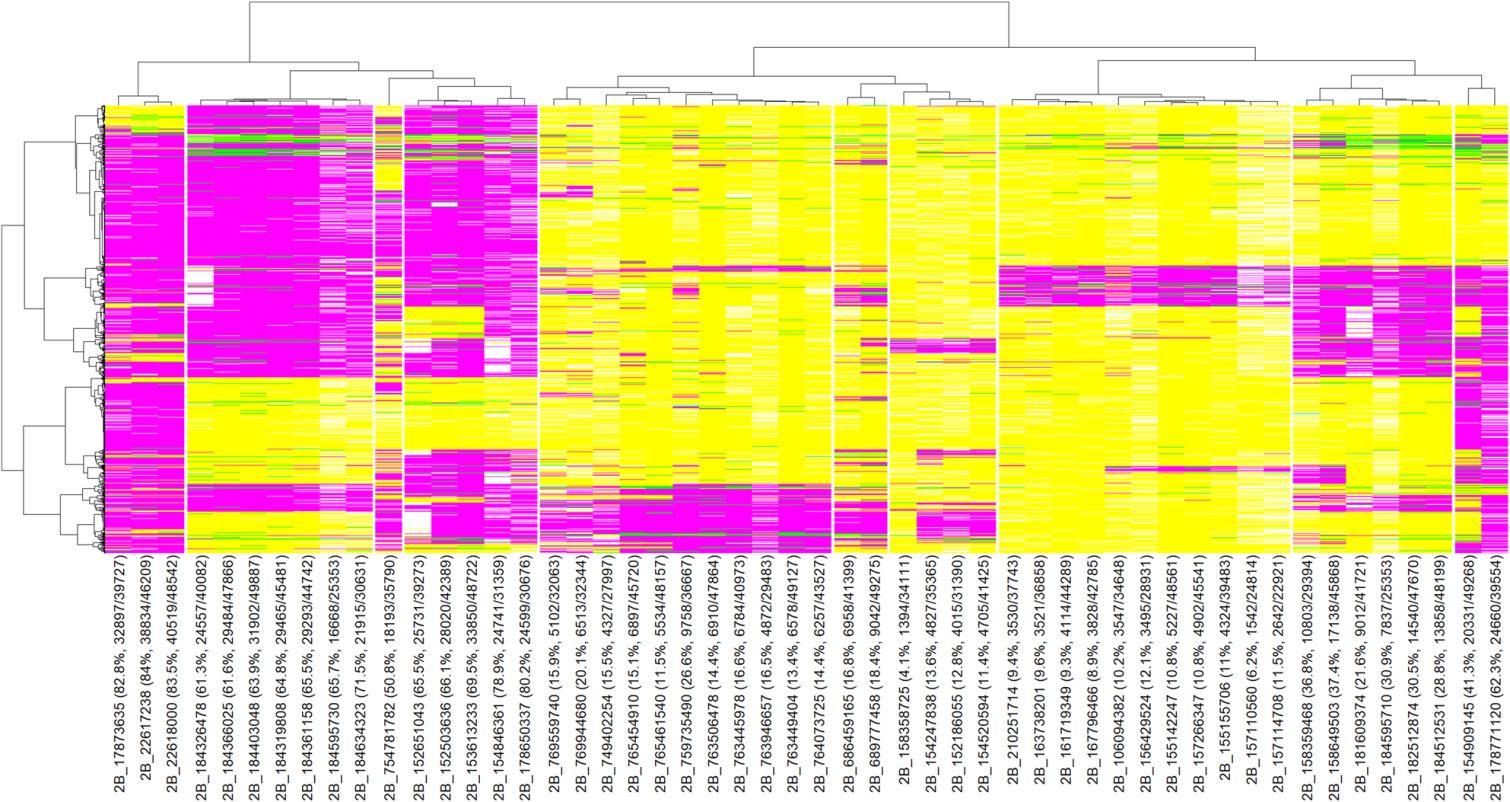
Allelic fingerprinting and clustering of stripe rust associated markers on chromosome 2B. The marker names are followed by the number of lines with the favorable alleles for the marker and the total number of non-missing alleles in parenthesis. The magenta color indicates the favorable allele (allele that has a decreasing effect on disease severity), the yellow color indicates the non-favorable allele (allele that has an increasing effect on disease severity), the green color indicates the heterozygote and the white color indicates missing data.

**Figure 6:**
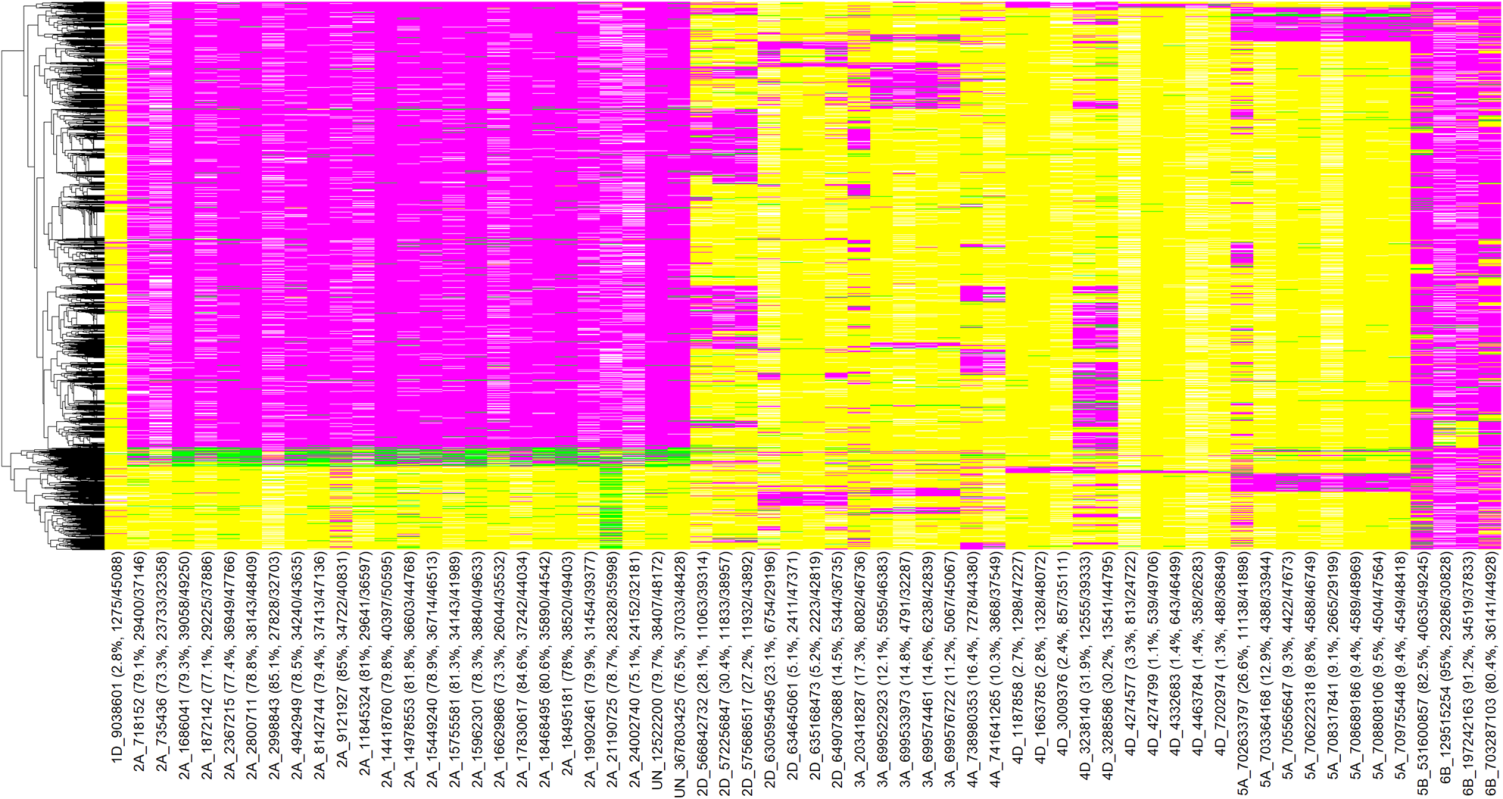
Allelic fingerprinting of stripe rust associated markers on chromosomes 1D, 2A, 2D, 3A, 4A, 4D, 5A, 5B, 6B and the unaligned markers. The marker names are followed by the number of lines with the favorable alleles for the marker and the total number of non-missing alleles in parenthesis. The magenta color indicates the favorable allele (allele that has a decreasing effect on disease severity), the yellow color indicates the non-favorable allele (allele that has an increasing effect on disease severity), the green color indicates the heterozygote and the white color indicates the missing data.

Allelic fingerprinting for the FAs, non-favorable alleles and heterozygous alleles was done for the 114 YR resistance associated repeatable markers (Figs. 5 and 6, Table S7 and S8) and for the 20 QTL (Table S9) in 52,067 CIMMYT wheat lines. Seven QTL including *QYr.cim-2AS.1, QYr.cim-2BS.1, QYr.cim-2BS.2, QYr.cim-5BL.1, QYr.cim-6BS.1* and *QYr.cim-6BL.1* had FAs between 68.9% and 94.2%, while all the other 13 QTL had FAs less than 38.5% (Table S8). For some markers where few lines carried FAs, the pedigrees were explored to identify the possible sources of the FAs. In *QYr.cim-1DS.1*, 917 (71.9%) of the 1,275 lines with FAs had SUPER 152 in the pedigree, despite SUPER 152 being heterozygous for the QTL. In *QYr.cim-2DL.2*, 1,661 (62.7%) of the 2,648 lines with FAs had Quaiu #1, Blouk #1 and Babax/Lr42//Babax in the pedigree, while 383 lines (14.5%) with FAs had Onix/Kbird and derived lines in the pedigree. In *QYr.cim-4DS.1*, 760 (59%) of the 1,287 lines had *Aegilops squarrosa* and 301 lines (23.4%) had Bavis, a synthetic wheat derived line. In *QYr.cim-5AL.1*, 2,481 (48.6%) of 5,101 lines had either Weebill1*2/Brambling or Brambling in the pedigree.

**Figure 7:**
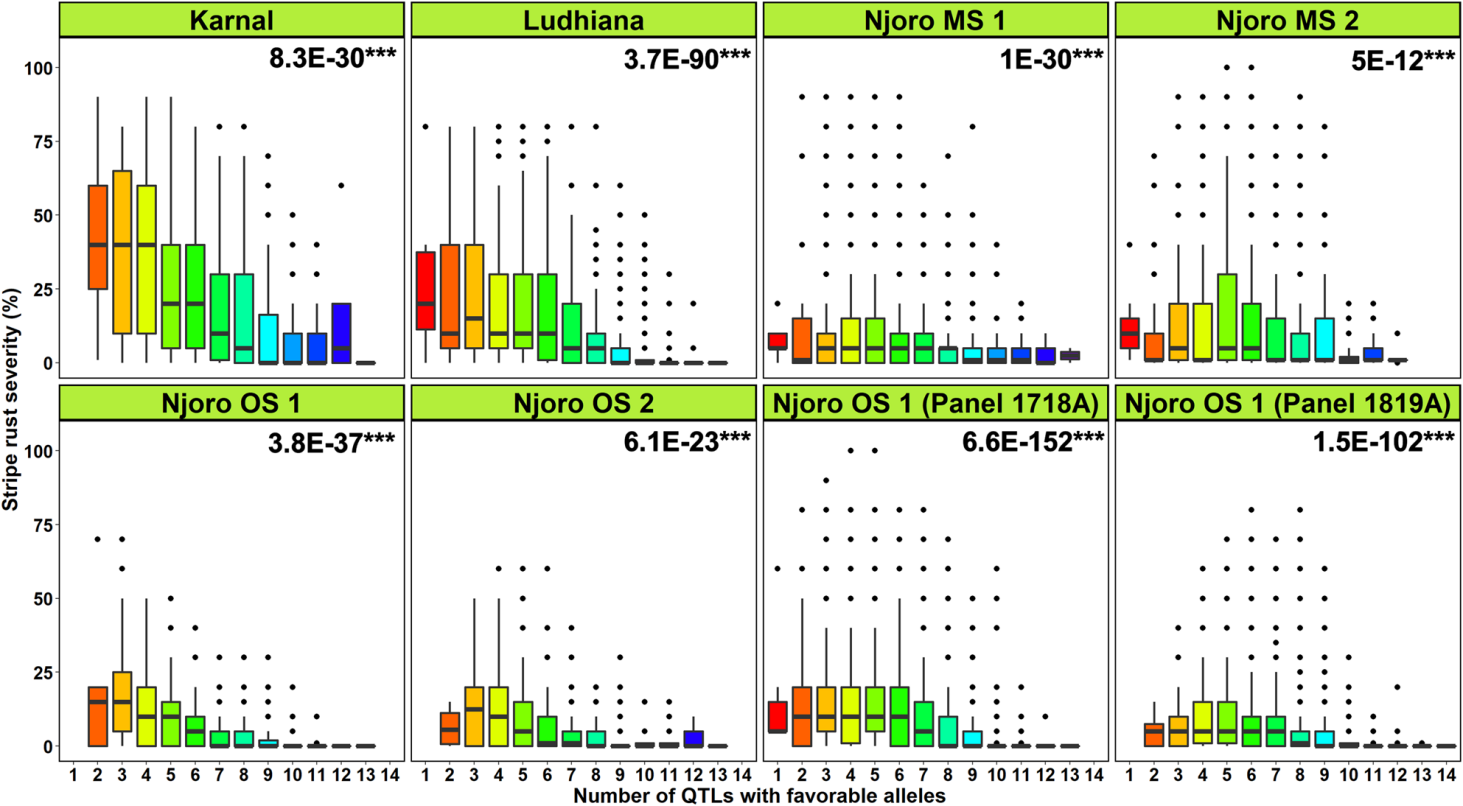
Number of QTL with favorable alleles plotted against the stripe rust severities of lines evaluated in different environments and panels. The QTL associated with more than one field resistance dataset were used and include *QYr.cim-1DS.1, QYr.cim-2AS.1, QYr.cim-2BS.1, QYr.cim-2BS.2, QYr.cim-2BS.3, QYr.cim-2BS.4, QYr.cim-2BS.5, QYr.cim-2BL.1, QYr.cim-2DL.1, QYr.cim-2DL.2, QYr.cim-3AL.1, QYr.cim-4AL.1, QYr.cim-4DS.1, QYr.cim-5BL.1, QYr.cim-6BS.1* and *QYr.cim-6BL.1*. The values on the top right of the panels indicate the two-sided *p *values for the test that there is no relationship between the number of QTL with favorable alleles and the stripe rust severities against the alternate hypothesis that there is a relationship. MS—main season and OS—off season.

To identify the lines that had high frequencies of FAs and may be deployed as potential parents, the lines carrying FAs in all the QTL and only in four QTL that were consistent across multiple datasets (*QYr.cim-2AS.1 QYr.cim-2BS.3 QYr.cim-2BS.4* and *QYr.cim-2DL.2*) were analyzed. Considering all the 20 QTL, line Pandora/Parula//2*Borlaug14 (GID8486516) had the maximum number of FAs (15). We also observed that 8.6% of the lines had FAs at 10–15 QTL, 77.5% of the lines had FAs at 5–9 QTL and 13.8% of the lines had FAs at 0–4 QTL. Considering only the four consistent QTL, 58 lines had FAs at all the four QTL, including 17 lines with the parent Quaiu*2/Kinde, and five lines with the parent Chen/Ae. sq//2*Opata/3/Tilhi/4/Attila/2*Pastor/5/Heilo/7/Kiskadee#1/5/Kauz*2/Mnv//Kauz/3/Milan/4/Bav92/6/Whear//2*Parula/2*Pastor. We also observed that about 3,550 lines (6.8%) had FA at three major QTL, 12,028 lines (23.1%) had FAs at two major QTL, 30,420 lines (58.4%) had FAs at one major QTL and 6,011 lines (11.5%) had no FAs at the major QTL. Furthermore, to understand the effect of having different numbers of QTL with FAs on the disease severity of lines, we regressed YR field severities in the India and Kenya panels against the number of QTL with FAs (only 17 FR QTL that were significant in more than one FR dataset were considered). Our results clearly indicated that the number of QTL in a line had highly significant relationships (at a *p *value threshold of 0.001) with the YR severities in all the datasets (Fig. 7). However, we also observed lines that had several QTL with FAs but had high disease severities and some lines that had few QTL with FAs and low severities, that can be attributed to not considering the effect size of the QTLs, imprecise phenotypes, insufficient marker coverage, rare QTL not detected in the study etc.

## Discussion

We performed comprehensive GWAS that provide excellent insights into the genetic architecture of YR field and seedling resistance, across multiple sites and years of evaluation in India, Kenya and Mexico. The 114 YR-associated repeatable GBS markers identified in this study have been anchored to the RefSeq v1.0, which will enable cross-comparisons across studies and will also serve as a valuable guide for genomics-assisted YR resistance breeding. Among the 20 QTL designated in this study, 13 were associated with only FR, 6 QTL were associated with both SR and FR (*QYr.cim-2AS.1, QYr.cim-2BL.1, QYr.cim-2BL.2, QYr.cim-2DL.2, QYr.cim-3AS.1* and *QYr.cim-4DS.2*) and one QTL (*QYr.cim-5AL.1*) was associated with only SR. To determine whether the QTL identified in this study were linked to known genes or previously reported QTL, we compared the positions of all the 20 QTL with previously reported genes/QTL with known positions in the RefSeq v1.0 and have only reported the QTL that were closely linked or are in the position of known QTL. Considering *QYr.cim-1DS.1* on chromosome 1DS, the known QTL in this region and their positions from *QYr.cim-1DS.1* include: (i) *QYrst.orr-1DS* from cultivar Stephens with the closest marker *wPt7946*^42^ located 7.4 Mb away (ii) *QYr.sun-1D* from the synthetic hexaploid CPI133872, with the closest marker *wmc147*^43^ located 8.8 Mb away (iii) *QYrdr.wgp-1DS* from the winter wheat cultivar Druchamp with the closest marker *IWA1789*^44^ located 0.4 Mb away (iv) *Qyr.wpg-1D.1* with the closest marker IWA6960^38^ at 8184870 bps located 0.85 Mb away (v) *QYr.ucw-1D* with the closest marker *IWA980*^45^ located 27.3 Mb away. Among these, *QYrdr.wgp-1DS* and *Qyr.wpg-1D.1* were the closest to *QYr.cim-1DS.1*.

On chromosome 2AS, *QYr.cim-2AS.1* was in the position of the 2NS translocation from *Aegilops ventricosa*, where the *Lr37/Sr38/Yr17* is located^46,47^. The gene *Yr17* is a race-specific resistance gene that was introgressed into the French wheat cultivar ‘VPM-1’ and widely used in the CIMMYT breeding program through lines derived from Mutus (Pedigree: Milan/S87230/4/Bow/Nac//Vee/3/Bjy/Coc) and Kachu (Pedigree: Kauz//Altar 84/Aos/3/Milan/Kauz/4/Vee/Koel), both of which have Milan in the pedigree^33,34^. However, *Yr17* was only effective against the races in Mexico and in the earlier Njoro panels (1314 and 1415). It was ineffective against the *Pst* races in Njoro since 2015–2016 and in India, while it has also been previously reported to be ineffective in Europe and other regions^21,48^. On chromosome 2BS, *QYr.cim-2BS.1* was 3.2 Mbs away from *Qyr.wpg-2B.1* linked to marker *IWA7370*^38^. The region between 152186055 and 184634323 bps where several markers with different FA frequencies were located is rich in YR genes^49,50^ and the known YR genes/QTL include: (i) *Yr31* gene from cultivars Pastor and Chapio flanked by marker *wPt-0079*^51,52^ at 187504047 bps (ii) *YrF* gene from cultivar Francolin #1 flanked by marker *gwm374* and *wmc474* at 165578329 and 172687189 bps, respectively (iii) *Yr41* gene close to marker *gwm374*^50^ at 165578329 bps (iv) *Qcim.2B.9* associated with YR in Ludhiana between 155142247 and 161419325 bps^33^ (v) *QYrlo.wpg-2BS* from cultivar Louise, linked to marker *wmc474* at 172687189 bps (vi) *QYr.caas-2BS* from Pingyuan flanked by markers *barc13* and *barc230*^53^ at 117249708 and 218506257 bps, respectively. While the exact number of QTL in this region could not be ascertained, it is possible that some of them indicate the *Yr31* gene present in CIMMYT lines with Pastor and Chapio in the pedigree and the *YrF* gene present in lines with Francolin #1. On chromosome 2BL, the most significant marker (2B_765461540) in *QYr.cim-2BL.2* associated with SR in most panels and FR in Karnal was 1.7 Mbs away from of the *Yr72* gene detected in wheat lines AUS 27506 and AUS 27894 flanked by the marker *IWB12294*^9^.

On chromosome 2DL, *QYr.cim-2DL.1* was associated with FR in the recent Njoro panels 1718 and 1819. The previously reported QTL for YR on chromosome 2DL included, (i) *QPst.jic-2D* from the UK wheat cultivar Guardian flanked by markers *Xgwm539* and *Xgwm349*^54^ that were at 513098578 and 629648566 bps respectively; (ii) a QTL from the Japanese cultivar Fukuho-komugi flanked by marker *Xgwm349*^55^; (iii) *Qyr.wpg-2D.2* flanked by marker *IWA5211* at 638147548 bps^38^ and (iv) *QPst.jic-2D* from German winter wheat cultivar Alcedo flanked by marker *gwm320* that was at 644277239 bps^56^. Among these, *QYr.cim-2DL.1* was in the interval of *QPst.jic-2D* from Guardian but might be indicating a novel QTL, as Guardian has not been used extensively in the CIMMYT breeding program, while 30% of the fingerprinted lines had FAs for this QTL. Another QTL, *QYr.cim-2DL.2* on chromosome 2DL that was associated with the highest number of datasets in India and Njoro in most of the recent evaluations involving panels 1718 and 1819 was 2.5 Mb away from the marker *wpt-667054* that was linked to the gene *Yr54*^57^. This gene was mapped from the CIMMYT spring wheat line QUAIU and the presence of the *QYr.cim-2DL.2* FAs in lines with Quaiu #1, Blouk #1 and Babax/Lr42//Babax all of which had Babax/Lr42//Babax in the pedigree affirms that this QTL refers to the *Yr54* gene that confers moderate resistance when present alone. While the effectiveness of this gene against races MEX96.11 and MEX08.13 in Mexico has been reported^57^, this study establishes its effectiveness to races in India (Karnal and Ludhiana) and Kenya (Njoro).

On chromosome 4AL, *QYr.cim-4AL.1* significant in three Ludhiana panels was in the same position as *Qcim.4A.5.3* reported to be associated with YR FR in Ludhiana^33^. The known YR resistance genes on chromosome 4AL include *Yr51* from wheat landrace, AUS27858, linked to marker *gwm160*^58^ that was 19.7 Mbs away from *QYr.cim-4A.1*. In addition, chromosome 4AL also had the *Yr60* gene from line Almop (Avocet*3//Lalbmono1*4/Pavon), whose closest marker *wmc313* (0.6 cM distal to *Yr60*)^59^ was 0.53 Mb away from marker 4A_738980353 in *QYr.cim-4A.1*. However, Lalbmono1*4/Pavon had the null allele for marker 4A_738980353 and was heterozygous for marker 4A_741641265 in our study and given that 15.9% of the fingerprinted lines had the FAs for *QYr.cim-4A.1*, while only 50 lines had Lalbmono1*4/Pavon in the pedigree, *QYr.cim-4A.1* might not be *Yr60*. Considering the QTL that have been reported at the distal end of chromosome 4AL, *QYr.wsu-4A.4* linked to markers *IWA4651* and *IWA3422*^60^ and *QYren.orz-4AL* linked to marker *wPt-1007*^61^, marker 4A_738980353 in *QYr.cim-4A.1* was only 0.54 Mb away from *IWA3422*, but it is unclear if they refer to the same QTL.

On chromosome 4DS, *QYr.cim-4DS.1* associated in the Njoro Panels 1718A and 1819A was 0.42 Mbs away from marker *BS00108770_51* linked to gene *Yr28* that originated from synthetic wheat and was also present in the synthetic-derived wheat line Soru#1^62,63^. The presence of FAs for *QYr.cim-4DS.1* in synthetic-derived wheat lines, confirms that *QYr.cim-4DS.1* indicates the *Yr28* gene. While the moderate resistance conferring gene *Yr28* is effective in relatively warm temperatures^63^, and has been reported to be largely ineffective when present alone^21,64^, it was significantly associated with YR resistance in Njoro in the two large panels used in this study and not in the smaller panels where the FA frequencies were less than 5%. On chromosome 5AL, *QYr.cim-5AL.*1 was in the position of the *Yr34/Yr48* gene flanked by marker *wPt-2873*^65–67^, 0.37 Mbs away from *QYr.caas-5AL* from Chinese landrace Pingyuan 50 linked to marker *Xbarc261*^53^ and it flanked the marker *IWA2558* linked to *QYrdr.wgp-5AL* from Druchamp^44^. However, most of these QTL were linked to APR and since, *QYr.cim-5A.*1 was only associated with SR, it could be a novel SR gene from Weebill1*2/Brambling. *QYr.cim-5BL.1* on chromosome 5BL was 2.8 Mb away from *QYrtb.orz-5BL* linked to marker *wPt-6105*^61^. On chromosome 6BS, *QYr.cim-6BS.1* flanked the known YR gene *Yr36* linked to marker *barc136*^68^ at 151377917 bps. But, the FAs at *QYr.cim-6BS.1* were present in a very high frequency (94.2%) in the fingerprinted lines and might not indicate *Yr36* that originated from *Triticum turgidum* ssp. d*icoccoides* and is present in only few CIMMYT lines. However, *QYr.cim-6BS.1* was 9.5 Mb away from the flanking marker for the APR gene *Yr78* (IWA4408)^69^ and might be indicating *Yr78* or another gene in that region.

It should also be highlighted that none of the durable pleiotropic APR genes (*Yr18, Yr29, Yr30* and *Yr46*) were detected in this study. While *Yr46* is absent in the germplasm we used, a diagnostic GBS marker for *Yr30* was not available. However, we used two markers on chromosome 1BL (1B_670207768 and 1B_670159907) that were significantly associated with YR in a biparental population (Apav/#1//Kenya Fahari/2*Kachu)^33^ and probably linked to the *Yr29* gene and observed FAs for the markers at very high frequencies in panels 1314–1819 (0.99 to 1), indicating that the gene is almost fixed in the CIMMYT germplasm. But this needs to be considered cautiously as the markers have not been validated in other populations and their LD with the* Yr29* gene is not known. Finally, we have also reported the YR associated allelic fingerprints for the largest panel of wheat breeding lines till date, that can be exploited by breeders to select parents, design strategic crosses for YR and eliminate lines with low to no FAs. The YR severities that could not be explained by the markers identified in this study, could be partly attributed to the fact that we have only used the 64% of markers that aligned to the RefSeq v1.0. While an impressive number of fingerprinted lines had FAs at ten and more YR QTL substantiating the multiple QTL resistance possessed by CIMMYT lines vs monogenic resistance, it also indicates plentiful opportunities for FA enrichment using molecular markers. The study is also of great significance for India where YR is a serious problem in the North Western plains zone comprising the states of Punjab and Haryana that have a major share in India’s wheat buffer stock. Overall, we hope that the results presented in this study will help broaden the understanding of the genetic architecture of YR resistance in different geographical regions, time-periods and wheat breeding lines and strengthen global YR resistance breeding efforts.

## Methods

### Populations

We used 23,346 advanced wheat breeding lines from CIMMYT that comprised eight different panels as follows: (i) Panels 1314, 1415, 1516, 1617 and 1718 with 1,092 lines each (ii) Panel 1819 with 1,228 lines (iii) Panel 1718A with 8,593 lines and (iv) Panel 1819A with 9,217 lines (Table S1). Panels 1718A and 1819A comprised the breeding lines from the first-year or stage 1 of yield testing that were developed using the selected-bulk breeding scheme as described in Juliana et al.^33^. Panels 1314, 1415, 1516, 1617, 1718 and 1819 included breeding lines from stage 2 of yield testing that were selected from stage 1 for high grain yield, good agronomic type, rust resistance and acceptable end-use quality.

### Evaluation of field resistance to YR

Field resistance to YR was scored as the percentage of infection (0–100%) on the plant. The lines were evaluated two to three times between the early- and late-dough stages, at 7 to 10-day intervals, with the first evaluation done after the severity of the susceptible checks reached 80–100%. In Karnal, India (29°41′N, 76°59′E), YR FR was evaluated for the lines in panel 1819 at the Indian Institute of Wheat and Barley Research during March 2019, using a mixture of four pathotypes namely: 46S119, 110S119, 47S103 and 110S84^70,71^. In Ludhiana, India (30°54′N, 75°51′E), we evaluated YR FR for the lines in panels 1314, 1415, 1617, 1718 and 1819 at the Borlaug Institute for South Asia during March–April 2014, March 2015, March 2017, March 2018 and March 2019, respectively. The predominant races collected from key cultivars like PBW343 (during 2013–2016) and HD2967 (during 2017 onwards) in Ludhiana including races 78S84, 46S119, 110S119 and 238S119 were used for inoculation.

In Njoro, Kenya (0°19′N, 35°56′E), YR FR was evaluated at the Kenya Agricultural and Livestock Research Organization during the main season (MS, June to October) as follows: (a) Panel 1314 during the second MS (MS2) in September 2014, (b) Panel 1415 during the first MS (MS1) in September 2014 (c) Panel 1516 during MS1 in September 2016 (d) Panel 1718 during MS1 and MS2, in September 2017 and 2018, respectively (e) Panel 1819 during MS1 in September 2018. Similarly, off season (OS, January to May) evaluations were done as follows: (a) Panel 1819 during the first off season (OS1) in April–May 2018 and the second off season (OS2) in April 2019, (b) Panel 1718A during OS1 in April–May 2018, (c) Panel 1819A during OS1 in April 2019. Evaluation was done under natural infection with the prevalent predominant races:* PstS2* in 2014 and 2015^72,73^,* PstS11*, followed by* PstS1* and* PstS2* in 2018 and 2019^74,75^.

In Celaya, Mexico (20°31′N 100°48′W), the lines in panel 1415 were evaluated for YR FR during March 2015 with the race Mex14.191^76^. In El Batan, Mexico (19°31′N 98°52′W), the lines in panel 1516 were evaluated for YR FR during August 2015 under natural infection with the race Mex14.191. In Toluca, Mexico (19°17′N, 99°11′W), the following panels were evaluated for FR to the Mexican *Pst* isolates including Mex96.11, Mex08.13 and Mex14.191 as described in Juliana et al.^34^: (a) Panel 1314 during August 2013, (b) Panel 1617 during August 2016 and (c) Panel 1819 during August 2018. A combined Toluca panel with all the 3,412 lines in panels 1314, 1617 and 1819 was also used for analyses.

### Evaluation of seedling resistance to YR in the greenhouse

The YR SR of lines in panels 1415, 1516, 1617, 1718 and 1819 to *Pst* race Mex14.191 was evaluated at CIMMYT’s greenhouses in El Batan, Mexico as described in Juliana et al.^34^. About 14 days post-inoculation, the YR infection types were recorded using a 0–9 scale^77^. We also used the data from all the 5,075 lines in the five panels as a combined seedling panel for analyses.

### Quality control of the phenotypic data

Quality control of the phenotypic data was done by removing outliers that were ‘K’ spreads from the center (‘K’ was assumed as 4) using the Huber’s robust fit outliers method^78^ in the JMP statistical software (https://www.jmp.com).

### Genotyping data and population structure analysis

The genotyping-by-sequencing (GBS) method^79^ was used to obtain genome-wide molecular markers and marker polymorphisms were called using the TASSEL (Trait Analysis by aSSociation Evolution and Linkage) version 5 GBS pipeline^80^. Marker discovery was done using a minor allele frequency of 0.01 and the resulting 6,075,743 GBS tags were aligned to the reference genome (RefSeq v1.0) assembly of the bread wheat variety Chinese Spring^81^ with an overall alignment rate of 64%. The tags were further filtered as described in Juliana et al.^33^ and 78,662 single-nucleotide polymorphisms were obtained. In each of the panels, the markers were filtered for: (i) Missing data less than 60%, (ii) Minor allele frequency greater than 0.05 and (iii) Heterozygosity less than 10% and the number of filtered markers in each panel is given in Table S1. In addition, we also filtered the lines in each panel for less than 50% missing data. Marker missing data was imputed using the LD-kNNi genotype imputation method^82^ in TASSEL version 5^83^. Population structure in all the panels was assessed using principal component analysis^84^.

### Genome‑wide association mapping and reference map with stripe rust associated markers

We performed genome-wide association studies for YR FR and SR in 28 datasets using the mixed linear model^85^ in TASSEL version 5. The mixed linear model was fitted using population structure as a fixed effect, accounted for by the first two principal components and kinship as a random effect, obtained by the centered identity-by-state method^86^. The optimum level of compression and the ‘population parameters previously determined’^87^ were used for running the mixed linear model. Correction for multiple testing was done using the Bonferroni method where an α level of 0.20 was used for declaring significant markers. The *p *values, additive effects and percentage variation explained by each marker were obtained and Manhattan plots were plotted using the CMplot ‘R’ package^88^. For constructing a reference map with the YR associated markers, we obtained all the markers that were significant at the 0.001 level across all the datasets. We then filtered only the repeatable markers that were significant in two or more datasets and aligned them to the RefSeq v1.0. Visualization of the reference map was done using Phenogram (https://visualization.ritchielab.org/phenograms/plot).

### Designation of linkage disequilibrium based quantitative trait loci

Linkage disequilibrium measures between the significant markers in each chromosome like the standardized disequilibrium coefficient (D′)^89^ and the correlation between alleles at the two marker loci (r^2^) were calculated in TASSEL, in addition to the *p *value for the existence of LD using the two-sided Fisher's Exact test. For LD estimation, the set of repeatable significant markers associated with YR and a large panel of 52,067 wheat lines (described below) were used. Any pair of markers with a D’ value greater than 0.80 and the *p *value for the existence of LD equal to 0 were designated into a LD-based QTL.

### Allelic fingerprinting and favorable allele frequencies

Allelic fingerprinting for YR resistance associated repeatable markers was done for 52,067 wheat breeding lines comprising 50,250 lines from the stage 1 of yield testing developed during 2013–2019, 1,385 lines from the international bread wheat screening nurseries developed during 2012–2014 and 432 lines from CIMMYT’s bread wheat crossing blocks. The allelic effects estimated from the mixed linear model were used in identifying the FA, defined as the allele that had a decreasing effect on YR severities or scores, the non-favorable (YR increasing) alleles and heterozygous alleles for each of the repeatable markers. In addition, the lines were also fingerprinted for the designated QTL using the most consistent marker alleles at those QTL. Heatmaps with the fingerprinted markers were obtained using the ‘R’ package ‘pheatmap’^90^. Furthermore, we fitted a linear regression model for the YR field severities in the India and Kenya panels using the number of QTL with FAs, considering only the 19 FR QTL that were significant in more than one FR dataset.

## Supplementary information

Supplementary information Fig. S1a

Supplementary information Fig. S1b

Supplementary information Fig. S1c

Supplementary information Fig. S1d

Supplementary information Fig. S1e

Supplementary information Fig. S2a

Supplementary information Fig. S2b

Supplementary information Fig. S2c

Supplementary information Fig. S2d

Supplementary information Fig. S2e

Supplementary information Fig. S2f

Supplementary information Fig. S2g

Supplementary information Fig. S2h

Supplementary file14 (XLSX 39301 kb)

## Data Availability

The stripe rust phenotyping data of 23,346 lines evaluated in different panels and environments is available in Supplementary Table 2.
