# Supplementary figures and images for: Genome-wide mapping and allelic fingerprinting provide insights into the genetics of resistance to wheat stripe rust in India, Kenya and Mexico
Source: Sci Rep. 2020 Jul 2;10:10908. doi: 10.1038/s41598-020-67874-x (PMC7331708; doi:10.1038/s41598-020-67874-x)

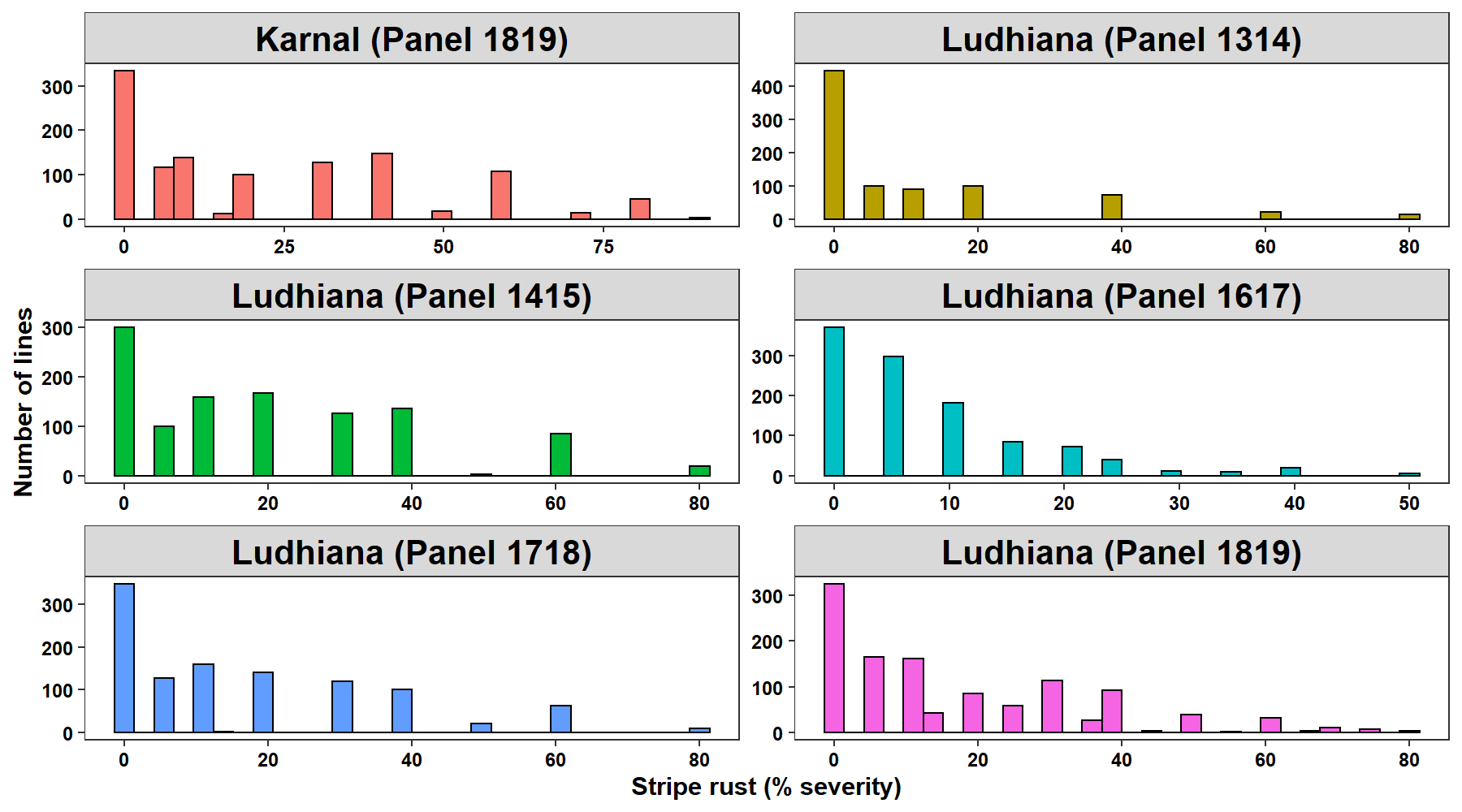

Supplement: Supplementary file 1 — Supplementary information Fig. S1a [file 41598_2020_67874_MOESM1_ESM.tiff]

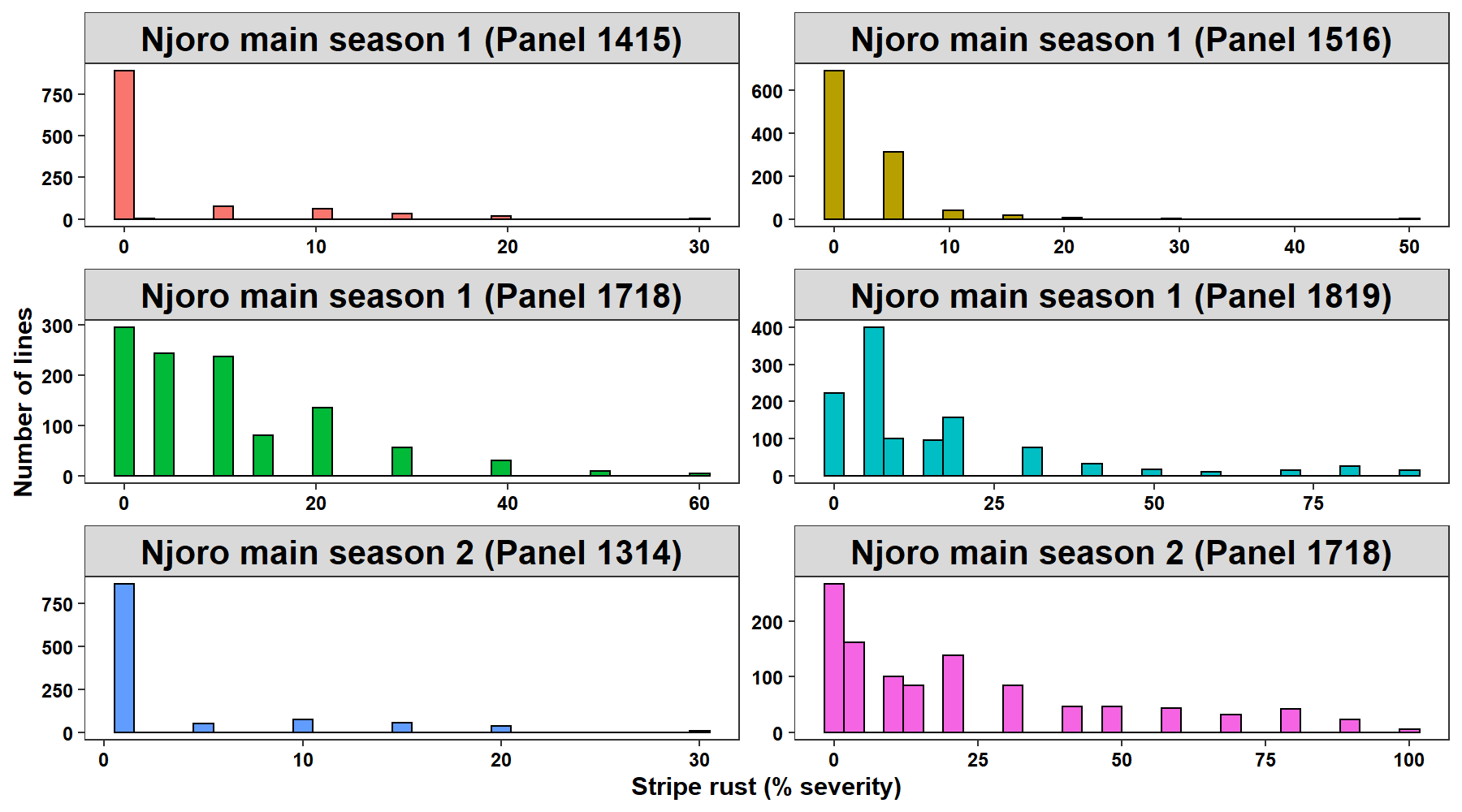

Supplement: Supplementary file 2 — Supplementary information Fig. S1b [file 41598_2020_67874_MOESM2_ESM.tiff]

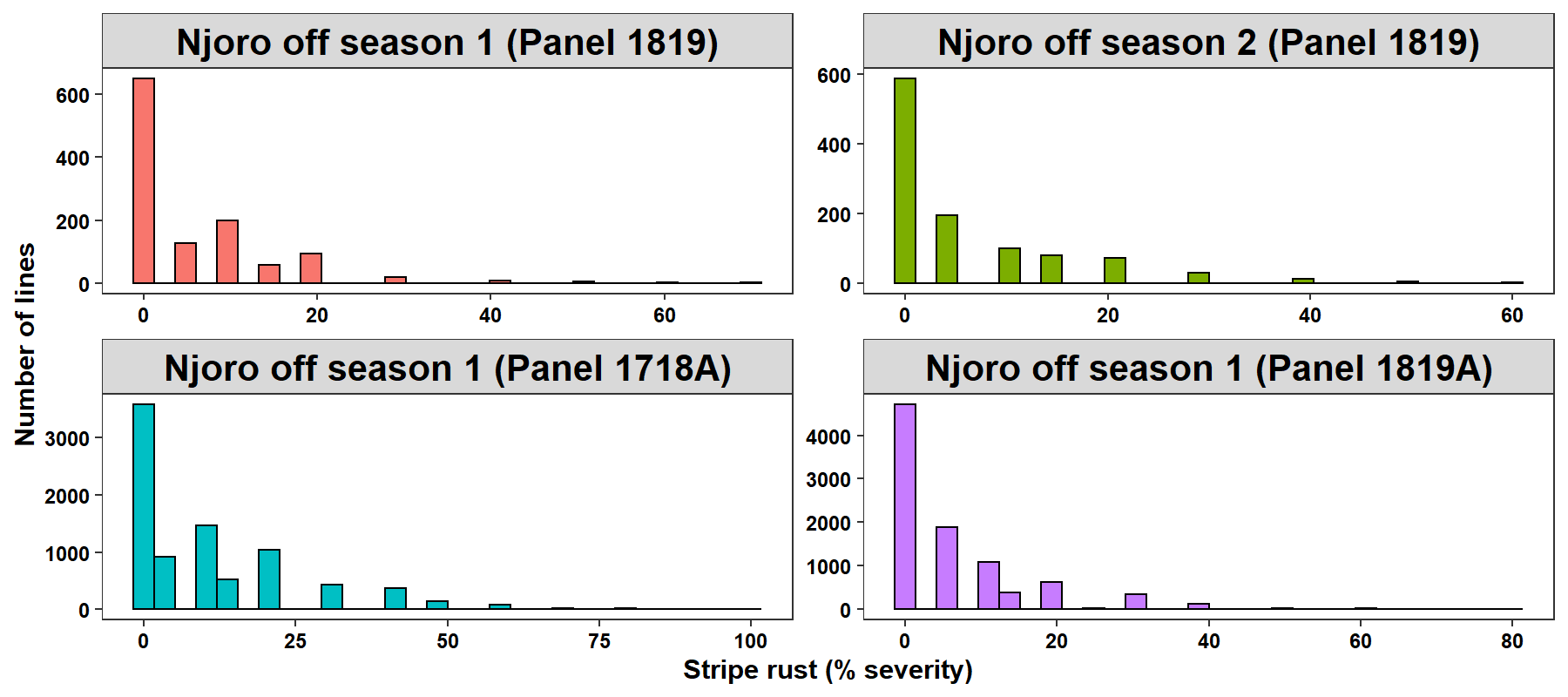

Supplement: Supplementary file 3 — Supplementary information Fig. S1c [file 41598_2020_67874_MOESM3_ESM.tiff]

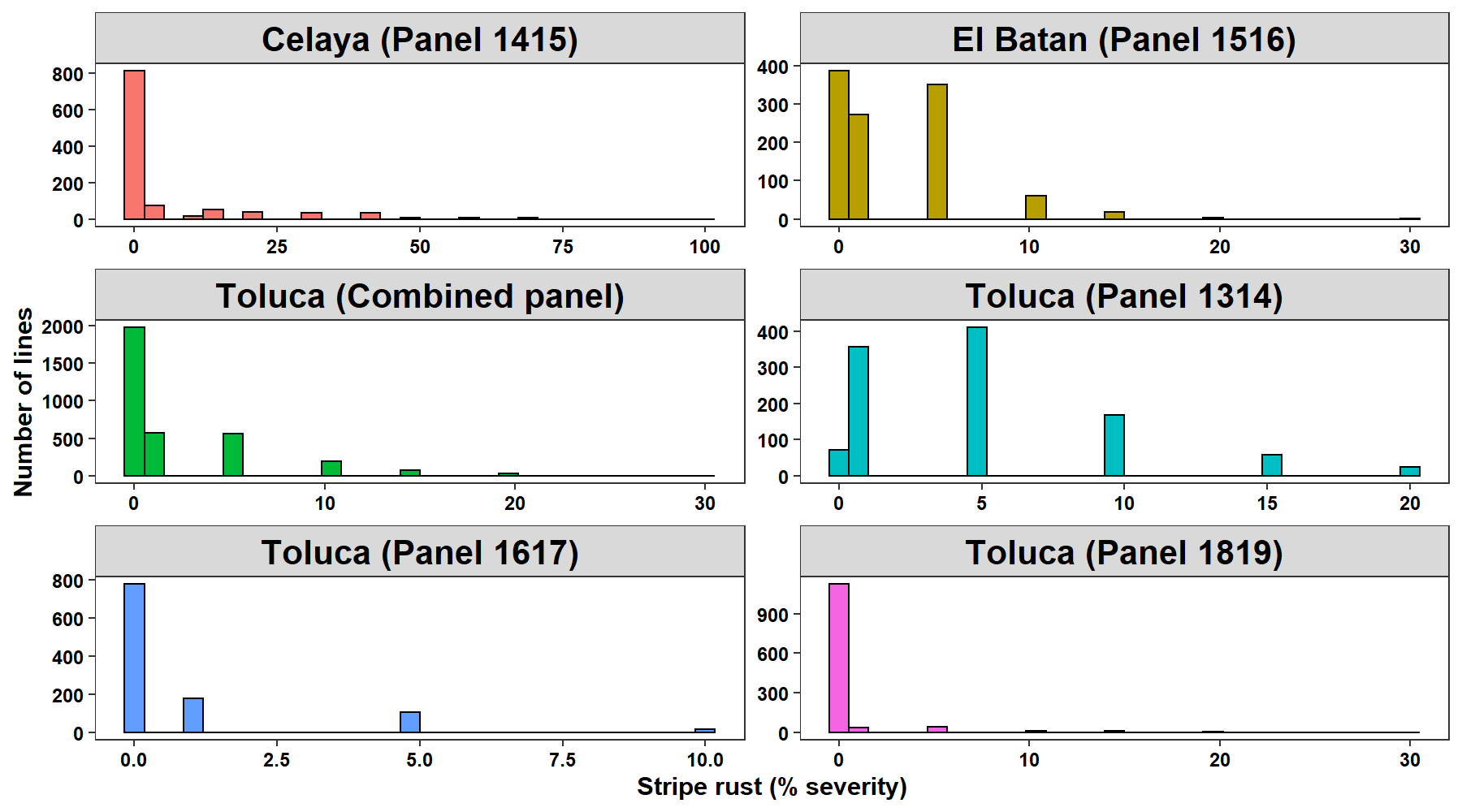

Supplement: Supplementary file 4 — Supplementary information Fig. S1d [file 41598_2020_67874_MOESM4_ESM.tiff]

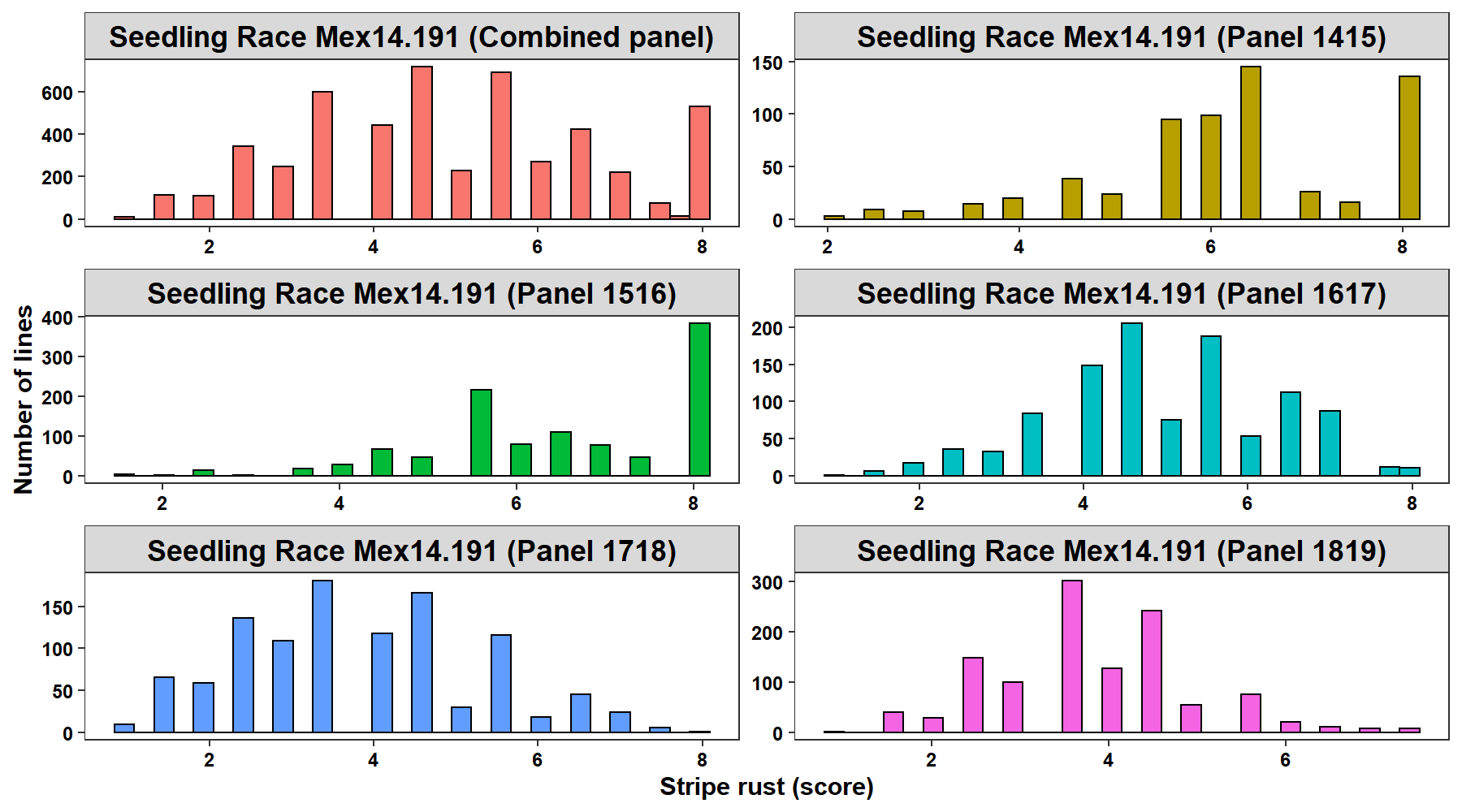

Supplement: Supplementary file 5 — Supplementary information Fig. S1e [file 41598_2020_67874_MOESM5_ESM.tiff]

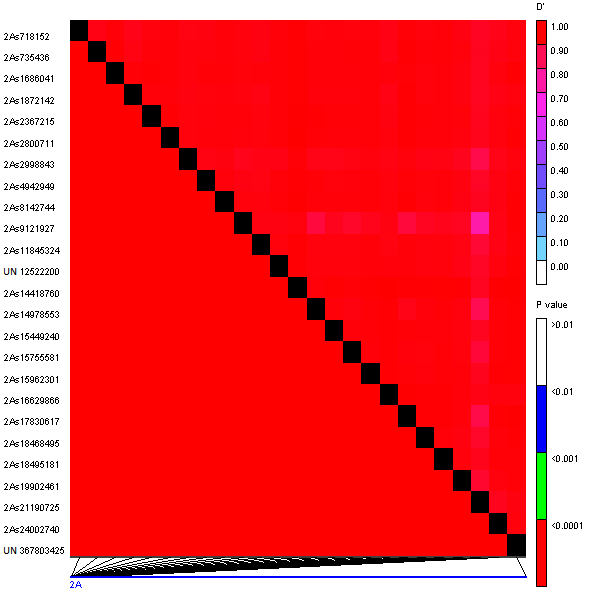

Supplement: Supplementary file 6 — Supplementary information Fig. S2a [file 41598_2020_67874_MOESM6_ESM.tiff]

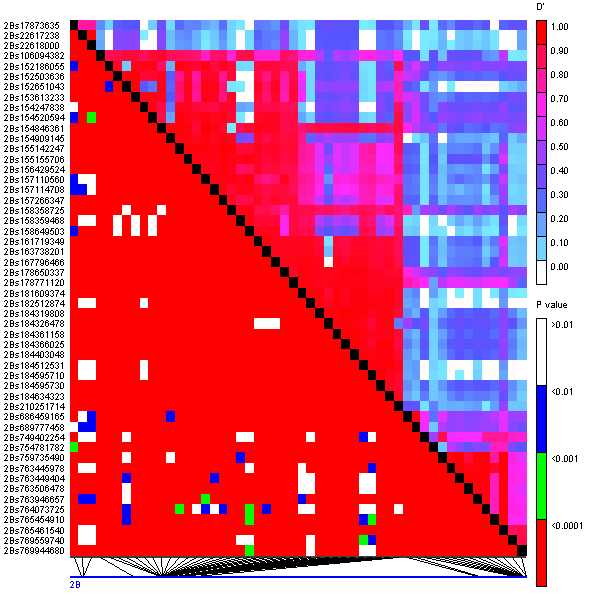

Supplement: Supplementary file 7 — Supplementary information Fig. S2b [file 41598_2020_67874_MOESM7_ESM.tiff]

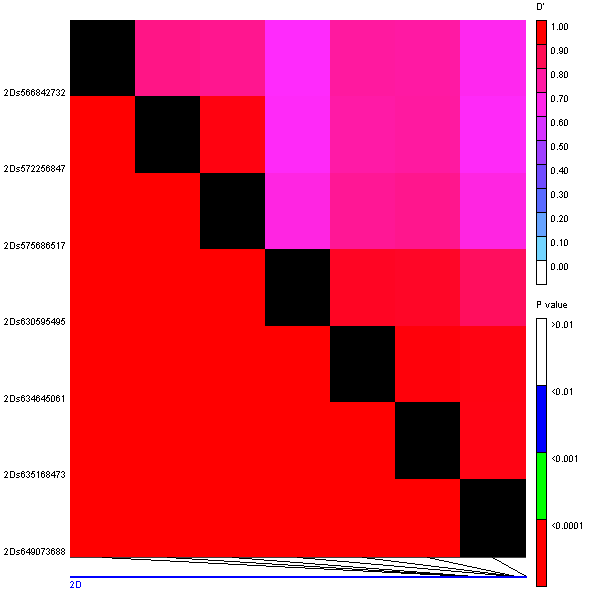

Supplement: Supplementary file 8 — Supplementary information Fig. S2c [file 41598_2020_67874_MOESM8_ESM.tiff]

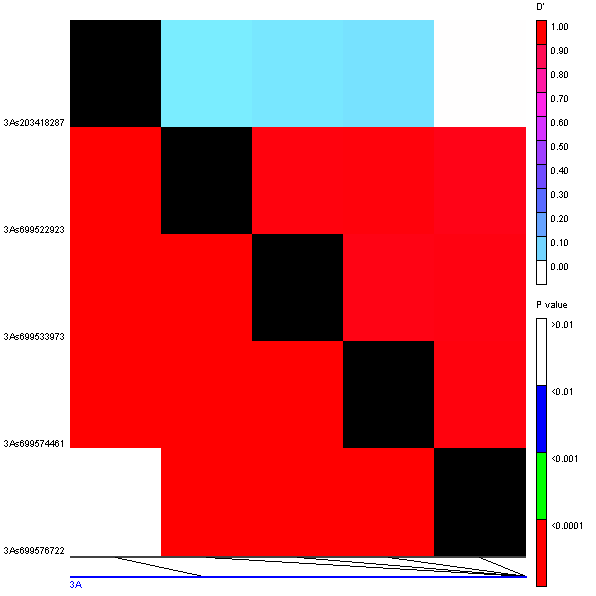

Supplement: Supplementary file 9 — Supplementary information Fig. S2d [file 41598_2020_67874_MOESM9_ESM.tiff]

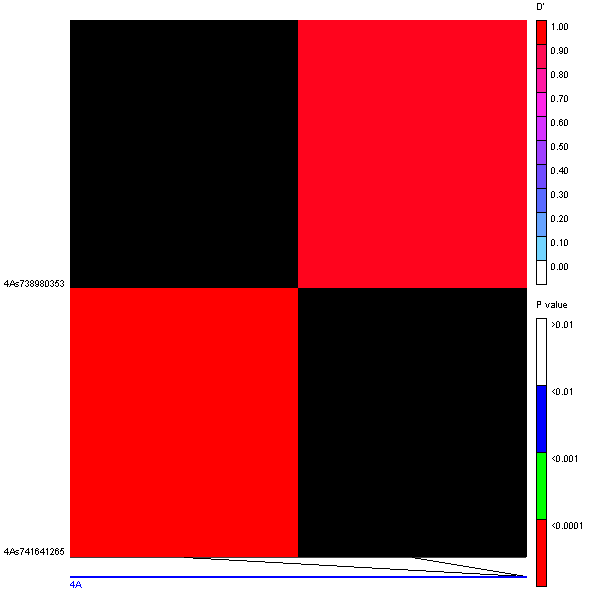

Supplement: Supplementary file 10 — Supplementary information Fig. S2e [file 41598_2020_67874_MOESM10_ESM.tiff]

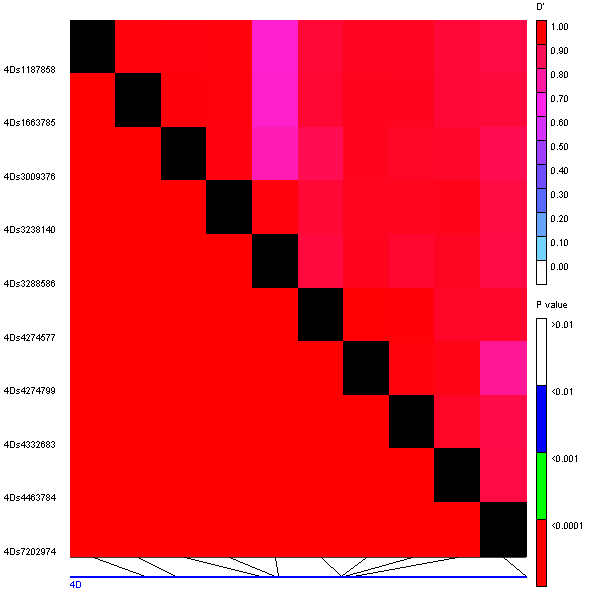

Supplement: Supplementary file 11 — Supplementary information Fig. S2f [file 41598_2020_67874_MOESM11_ESM.tiff]

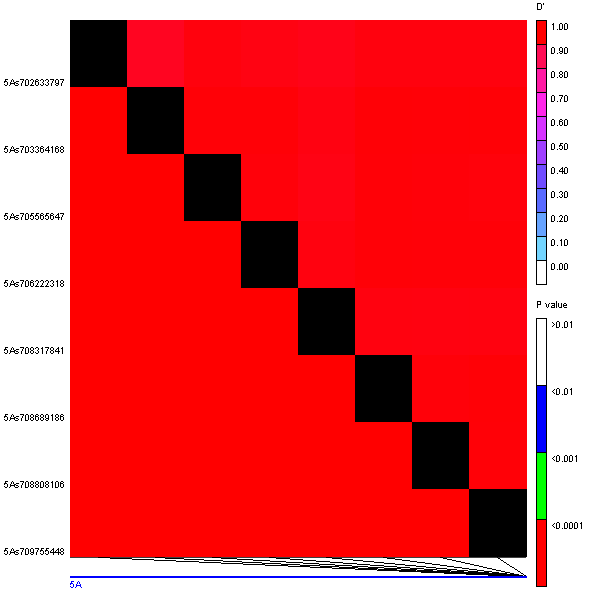

Supplement: Supplementary file 12 — Supplementary information Fig. S2g [file 41598_2020_67874_MOESM12_ESM.tiff]

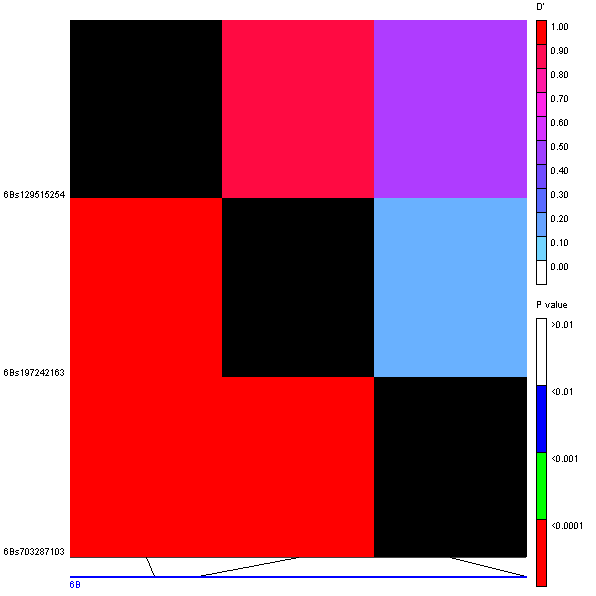

Supplement: Supplementary file 13 — Supplementary information Fig. S2h [file 41598_2020_67874_MOESM13_ESM.tiff]
